# Commercialized artemisinin derivatives combined with colistin protect against critical Gram-negative bacterial infection

**DOI:** 10.1038/s42003-022-03898-5

**Published:** 2022-09-08

**Authors:** Yonglin Zhou, Baichen Liu, Xiuling Chu, Jianqing Su, Lei Xu, Li Li, Xuming Deng, Dan Li, Qianghua Lv, Jianfeng Wang

**Affiliations:** 1 Department of Respiratory Medicine, Center for Pathogen Biology and Infectious Diseases, Key Laboratory of Organ Regeneration and Transplantation of the Ministry of Education, The First Hospital of Jilin University, Changchun, 130021 Jilin China; 2 State Key Laboratory for Zoonotic Diseases, College of Veterinary Medicine, Jilin University, Changchun, 130062 Jilin China; 3 The Second Bethune Clinical Medical College of Jilin University, Changchun, 130012 Jilin China; 4 College of Agronomy, Liaocheng University, Liaocheng, 252000 Shandong China

**Keywords:** Antimicrobial resistance, Transcriptomics, Bacterial infection

## Abstract

The emergence and spread of the *mcr-1* gene and its mutants has immensely compromised the efficient usage of colistin for the treatment of drug-resistant Gram-negative bacterial infection in clinical settings. However, there are currently no clinically available colistin synergis. Here we identify artemisinin derivatives, such as dihydroartemisinin (DHA), that produces a synergistic antibacterial effect with colistin against the majority of Gram-negative bacteria (FIC < 0.5) without induced resistance, particularly those carrying the *mcr-1* gene. Mechanism analysis reveals the direct engagement of DHA with the active center of MCR-1 to inhibit the activity of MCR-1. Meanwhile, the results from transcriptome and electron microscope analysis show that DHA could also simultaneously affect the flagellar assembly and the energy metabolism of bacteria. Moreover, in the mouse infection models of Gram-negative bacteria, combination therapy shows remarkable treatment benefits, as shown by an improved survival rate, reduced morbidity, alleviated pathological injury and decreased bacterial loading. Due to the generally safe profile of specialized malaria medication administration in humans, artemisinin derivatives are a promising class of multi-target inhibitors on bacterial resistance and virulence that can be used to extend the usage life of colistin and to tackle the inevitability of serious bacterial infection with colistin.

## Abstract (web summary)

The anti-malaria drugs artemisinin derivatives produce a synergistic antibacterial effect with colistin against Gram-negative bacteria by simultaneously inhibiting MCR-1 function and hindering flagella assembly and energy metabolism.

## Introduction

The progressive global increase in microbial resistance, especially the discovery of the plasmid-mediated mobile colistin resistance genes, *mcr-1* and its mutants, is now worrisome and has led global scientific research institutions and governments to unify the international agenda to combat complex microbial resistance infections^1,2^. MCR-1, which has been found in the most common and critical Gram-negative bacteria, including *Enterobacteriaceae* (such as *Escherichia coli* (*E. coli*), *Klebsiella pneumonia* (*K. pneumonia*) and *Salmonella typhimurium* (*S. typhimurium*)), *Acinetobacter baumannii* (*A. baumannii*) and *Pseudomonas aeruginosa*, is a novel phosphoethanolamine transferase with a high ability to hinder polymyxins interacting with lipid A^3,4^. Therefore, there is an urgent need to seek a potent MCR-1 inhibitor to limit the induction of polymyxin resistance and aid in infectious diseases caused by the “Superbug”.

The virulence and resistance of bacteria are equally critical in the treatment of bacterial infections^5^. The ability to precisely regulate the expression of virulence factors is principal for pathogenic bacteria to successfully colonize a host. Flagellum-driven motility is critical for many pathogens, such as *S. typhimurium* and *Clostridium difficile*, to establish productive infections^6^. And flagellar assembly is related to the secretion of effector proteins by the type 3 secretion system^7,8^. In addition, it is inseparable from energy metabolism in the process of virulence factor synthesis, export or the secretion of bacteria.

The development pathway for new antibacterial drugs, such as designing new structures and finding new targets, is however time-consuming and costly. To address this important issue, conventional drugs used in a new manner are a shortcut to rapidly response to bacterial resistance. Artemisinin is a “special gift” to millions of malaria patients worldwide from the Chinese Nobel Laureate Youyou Tu and her research group^9,10^. In recent years, research teams from all over the world, especially from China, have been exploring and developing a variety of artemisinin derivatives with significant clinical effects against malaria, which have been put into human clinical use, including dihydroartemisinin (DHA), artemether, artesunate and arteether, with oral tablets and injectable dosage forms^11–13^. Artemisinin derivatives can fully satisfy different routes of drug delivery, and the significant effect of artemisinin derivatives in the treatment of malaria is attributed to their multiple dosage forms and good absorption effect. It has also been found to be effective in treating diseases such as lupus erythematosus^14–16^. Our study confirmed the effects of artemisinin derivatives such as DHA simultaneously inhibiting MCR-1 function to restore the antimicrobial activity of colistin and mitigate the virulence of critical Gram-negative bacteria mediated by flagellar assembly. Furthermore, dihydroartemisinin with lower negative effects of the decrease in hemoglobin, is the metabolite of artesunate and artemether in vivo, and thus, this study mainly focuses on dihydroartemisinin as the core drug.

## Results

### Artemisinin derivatives showed synergies in combination with colistin against Gram-negative bacteria, especially MCR-1-positive *Enterobacteriaceae*

**Fig. 1:**
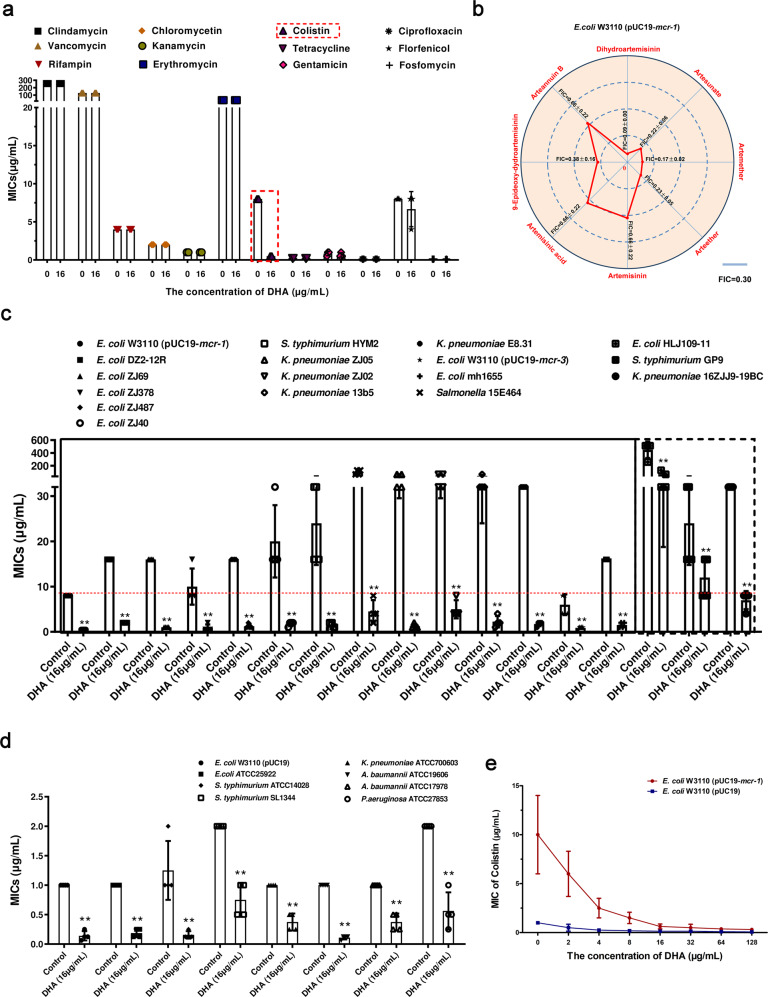
DHA restores the antibacterial activity of colistin. **a** DHA (16 μg/mL) only had a significant synergistic effect with colistin (outlined in red) among 12 different antibiotics against *E. coli* W3110 (pUC19-*mcr-1*) (*n* = 3). **b** Contribution capacity chart of FIC showing that artemisinin derivatives such as artesunate, artemether, arteether and 9-epideoxy-dydroartemisinin also had a significant synergistic effect with colistin against *E. coli* W3110 (pUC19-*mcr-1*) (*n* = 4). **c**–**e** DHA restored the antibacterial activity of colistin against MCR-positive bacteria (outlined in solid), MCR-negative polymyxin-resistant bacteria (outlined in dashed) and polymyxin-sensitive bacteria. MICs of colistin without DHA for all the tested bacteria was used as control. Data are representative of four independent experiments. **P < 0.01.

Our previous study showed that some active components of traditional Chinese herbs had a synergistic effect with polymyxins against MCR-1-positive bacterial strains^17,18^. However, almost none of them have commercialized formulations for rapid clinical application. In view of this urgent need, we applied the method of checkerboard MIC assays to screen polymyxin synergists from commercialized active components of traditional Chinese herbs. The results from the assay applying the checkerboard MIC successfully confirmed the synergistic effect between only colistin and DHA in the strain of *E. coli* W3110 (pUC19-*mcr-1*) (FIC = 0.09 ± 0.00) in the presence of 16 µg/mL DHA, whereas no synergy was observed with any of the other tested antibiotics (Fig. 1a, Supplementary Fig 1 and Supplementary Table 1). In addition, other artemisinin derivatives such as artesunate, artemether and arteether also restored the antibacterial effect of colistin against *E. coli* W3110 (pUC19-*mcr-1*) (FIC < 0.25) (Fig. 1b and Supplementary Fig 2). Excitingly, a synergistic effect between DHA and colistin was also observed in all the tested polymyxin-resistant strains and polymyxin-sensitive strains, except for an MCR-negative polymyxin-resistant isolate *S. typhimurium* GP9 (FIC = 0.53 ± 0.00) (Fig. 1c–e, Supplementary Figs. 3–5 and Supplementary Table 1).

**Fig. 2:**
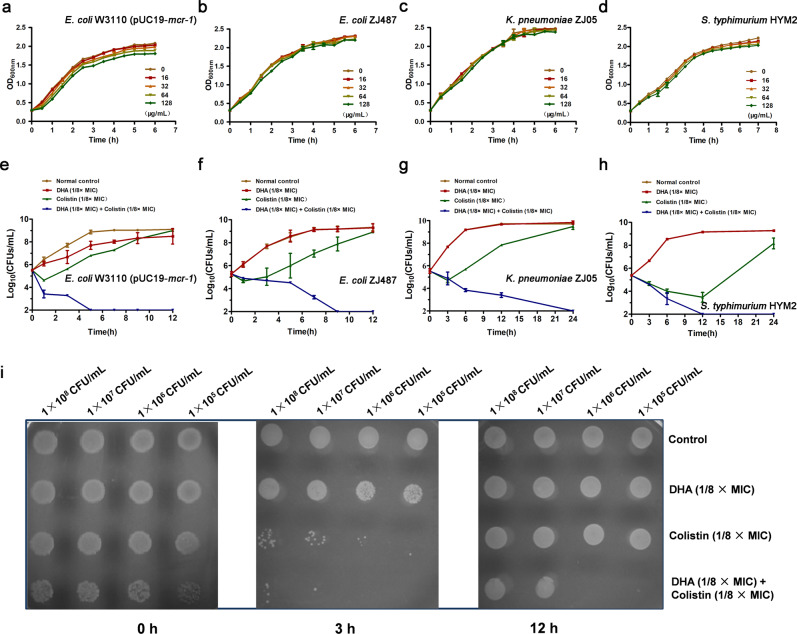
DHA enhances the bactericidal efficacy of colistin without affecting bacterial growth. **a**–**d** Growth curves for *E. coli* W3110 (pUC19-*mcr-1*) (**a**), *E. coli* ZJ487 (**b**), *K. pneumoniae* ZJ05 (**c**) and *S. typhimurium* HYM2 (**d**) cultured in the presence of various concentrations of DHA (0, 16–128 µg/mL). **e**–**h** Time-killing curves for DHA, colistin, combination and control treatment against *E. coli* W3110 (pUC19-*mcr-1*) (**e**), *E. coli* ZJ487 (**f**), *K. pneumoniae* ZJ05 (**g**) and *S. typhimurium* HYM2 (**h**). Values represent the averages of three independent experiments. **i** Three microliters of *E. coli* W3110 (pUC19-*mcr-1*) bacteria with different treatments (concentrations from 1 × 10^8^ CFU/mL to 1 × 10^5^ CFU/mL) were dropped on LB agar plates at 0, 3 and 12 h and cultured at 37 °C overnight. All the growth curves and bactericidal test were determined in triplicate, and representative data are displayed.

The results from the growth curve showed that none of the concentrations of DHA (16-128 µg/mL) affected the growth of MCR-1-positive *E. coli* W3110 (pUC19-*mcr-1*), *E. coli* ZJ487, *K. pneumoniae* ZJ05 and *S. typhimurium* HYM2 (Fig. 2a–d). In contrast, the combination of colistin and DHA resulted in the elimination of *E. coli* W3110 (pUC19-*mcr-1*), *E. coli* ZJ487, *K. pneumoniae* ZJ05 and *S. typhimurium* HYM2 at 5, 9, 24 and 5 h post-administration, respectively (Fig. 2e–h). This synergistic bactericidal effect was also validated in an agar plate germicidal test in which the combination completely killed ≤1 × 10^6^ CFU/mL of *E. coli* W3110 (pUC19-*mcr-1*) dropped on the plate at 12 h (Fig. 2i).

**Fig. 3:**
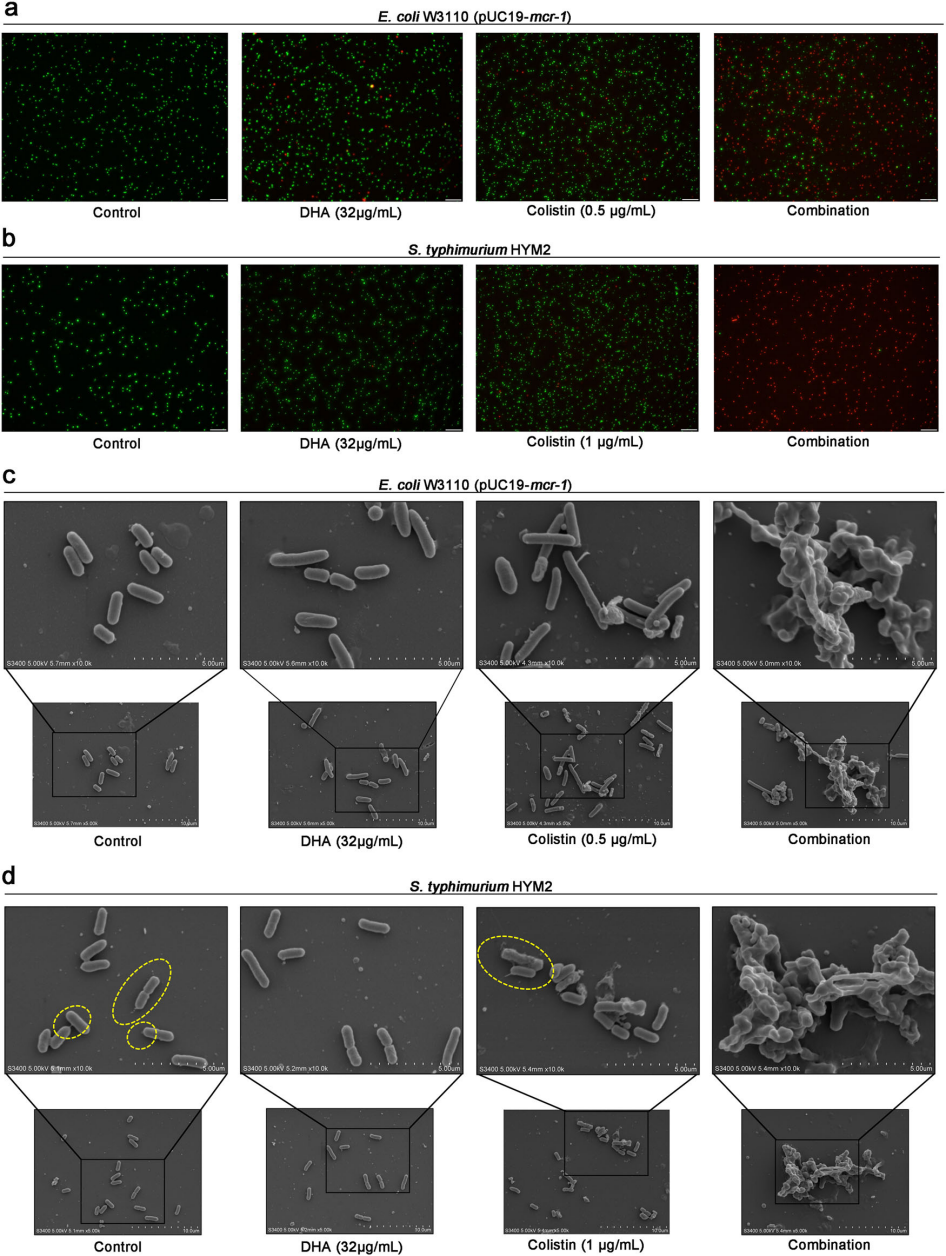
DHA in combination with colistin effectively kills the tested bacteria. **a**, **b** Fluorescence labeling analysis for *E. coli* W3110 (pUC19-*mcr-1*) (**a**) and *S. typhimurium* HYM2 (**b**) were treated with DHA (32 µg/mL), colistin (0.5/1 µg/mL) the combination or control (bacteria without any treatment), and live bacteria were dyed green and dead bacteria were dyed red (scale bar = 50 μm). **c**, **d** SEM analysis (scale bar = 5 μm) for *E. coli* W3110 (pUC19-*mcr-1*) (**c**) and *S. typhimurium* HYM2 (**d**) was also performed with the above treatment (5.00 kV, bar = 5 μM, 10 μM). Only in the control group and 1 μg/mL colistin treatment group observed the filamentous flagella at both ends of the *S. typhimurium* (yellow dashed circles). The experiments were repeated three times independently, and representative data are displayed.

**Fig. 4:**
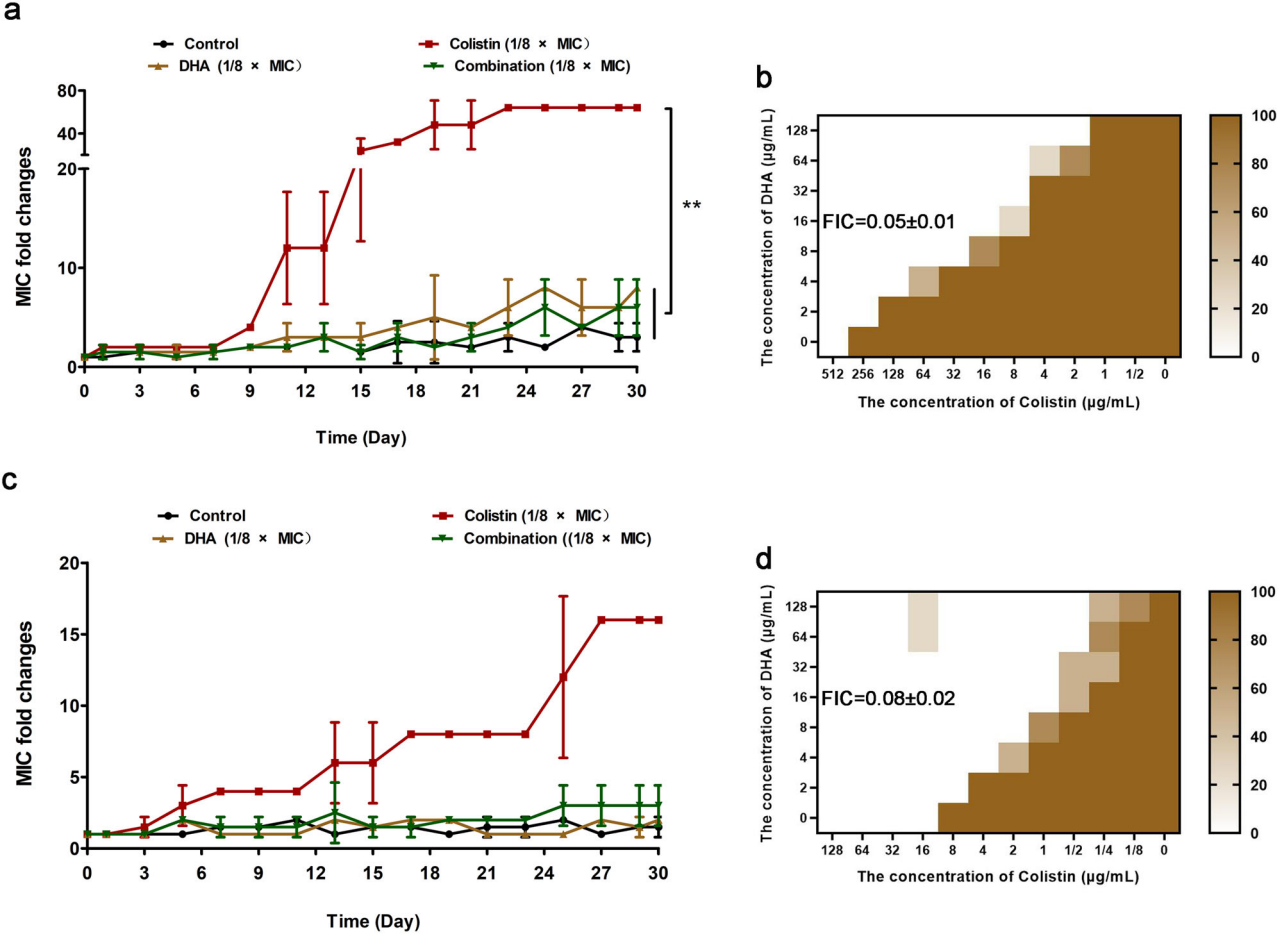
DHA (1/8 × MIC) does not induce the resistance of bacteria to colistin. **a**, **c** MIC fold changes of 1/8 × MIC DHA, 1/8 × MIC colistin or 1/8 × MIC combination (the concentration of DHA was 16 µg/mL) of *E. coli* W3110 (pUC19-*mcr-1*) (**a**) and *E. coli* W3110 (pUC19) (**c**). **b**, **d** The checkerboard MIC of *E. coli* W3110 (pUC19-*mcr-1*) (**b**) and *E. coli* W3110 (pUC19) (**d**) following 1/8 × MIC colistin induction was determined after day 30. All checkerboard MICs were determined in quadruplicate, and used the color shade to indicate the probability of bacterial growth in the well. The more bacterial growth visible to the naked eye, the darker the color. The concentration of DHA was 16 µg/mL calculated for FIC. **P < 0.01.

In agreement with the above bactericidal test, the combination therapy led to a change in bacterial morphology, visible shrinkage of the cell wall and gathering to death of *E. coli* W3110 (pUC19-*mcr-1*) and *S. typhimurium* HYM2, as evident by noticeably more dead bacteria (red dyed) (Fig. 3a, b) and visibly destroyed bacteria under SEM (Fig. 3c, d). Interestingly, compared with the control or colistin treatment, the flagella of *S. typhimurium* HYM2 disappeared after DHA (32 μg/mL) treatment, as observed by SEM (Fig. 3d). In addition, the results of the serial passage colistin-resistant test showed that 1/8 × MIC colistin could induce *E. coli* W3110 (pUC19-*mcr-1*) and *E. coli* W3110 (pUC19) resistance to colistin, compared with the 1/8 ×  MIC DHA, 1/8 × MIC combination and control (Fig. 4a, c). Notably, DHA could also restore the antibacterial effect of colistin after serial passage induction of colistin (Fig. 4b, d), suggesting that DHA may extend the clinical usage life of polymyxins. Taken together, our results suggested that artemisinin derivatives, such as DHA may significantly restore the antibacterial activity of polymyxins through multitarget interactions.

### DHA robustly protects host cells from preventing pathogen invasion

**Fig. 5:**
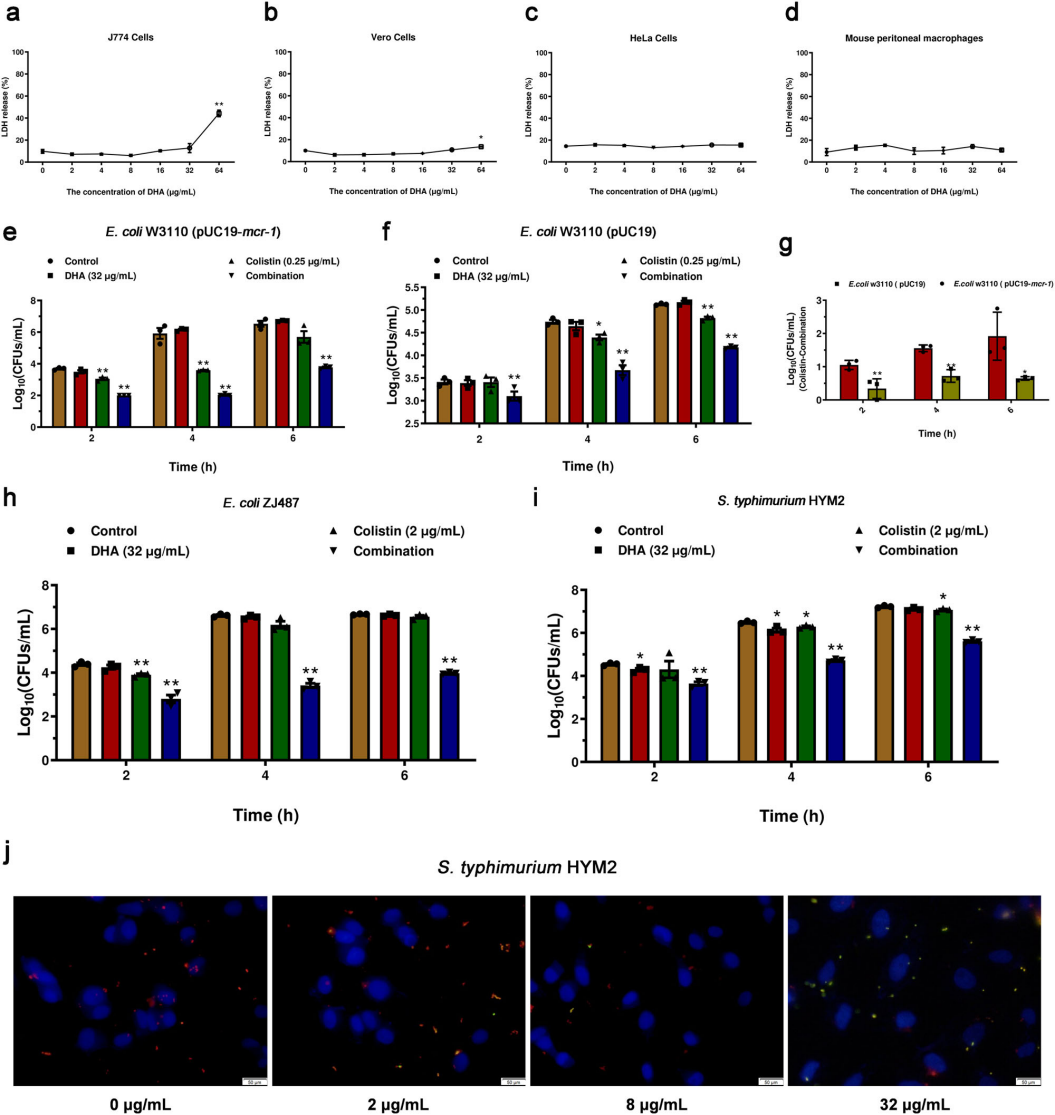
DHA alone or in combination with colistin protects cells from preventing pathogen invasion. **a**–**f**, **h**, **i** LDH release by J774 cells (**a**), Vero cells (**b**), HeLa cells (**c**) and mouse peritoneal macrophages (**d**) co-cultured with various concentrations of DHA (0, 2–64 µg/mL). The invasion of *E. coli* W3110 (pUC19-*mcr-1*) (**e**), *E. coli* W3110 (pUC19) (**f**) and *E. coli* ZJ487 (**h**) in Vero cells and *S. typhimurium* HYM2 (**i**) in HeLa cells was determined at 2 h, 4 h and 6 h (*n* = 3). **g** The abilities between colistin and the combination to inhibit the invasion of *E. coli* W3110 (pUC19-*mcr-1*) and *E. coli* W3110 (pUC19) were calculated (Log_10_(The number of invasive bacteria after treatment with colistin)-Log_10_(The number of invasive bacteria after treatment with the combination)) at 2, 4, and 6 h. **j** Immunofluorescence microscopy detection (scale bar = 50 μm) of DHA on *S. typhimurium* HYM2 invasion of HeLa cells showed that the number of intracellular bacteria was lower (red dyed) as the concentration of DHA increased. It showed that red-entered cells became less. *P < 0.05. **P < 0.01.

We further evaluated the safety of DHA on different cells including J774 cells, Vero cells, HeLa cells and mouse peritoneal macrophages. DHA showed no cytotoxicity to all the tested cells at low concentrations no more than 32 μg/mL at 6 h (IC_50_ > 64 μg/mL) (Fig. 5a–d). Furthermore, DHA combined with colistin can significantly prevent *E. coli* (including *E. coli* W3110 (pUC19-*mcr-1*), *E. coli* W3110 (pUC19) and *E. coli* ZJ487) from invading Vero cells and *S. typhimurium* HYM2 from invading HeLa cells at 2, 4 and 6 h (Fig. 5e–i). In addition, better protection was observed for MCR-1-carrying strains (Fig. 5g). However, DHA treatment hindered *S. typhimurium* HYM2 invading HeLa cells but not *E. coli* strains invading Vero cells by invasion assay and immunofluorescence microscopy, which may be due to impeding the flagellar assembly of *S. typhimurium* by DHA (Fig. 5i, j and Supplementary Fig. 6a). Thus, DHA alone or in combination with colistin protected different cells from bacterial-mediated damage at nontoxic concentrations.

### Engagement of DHA with the active center of MCR-1 inhibits MCR-1 activity

**Fig. 6:**
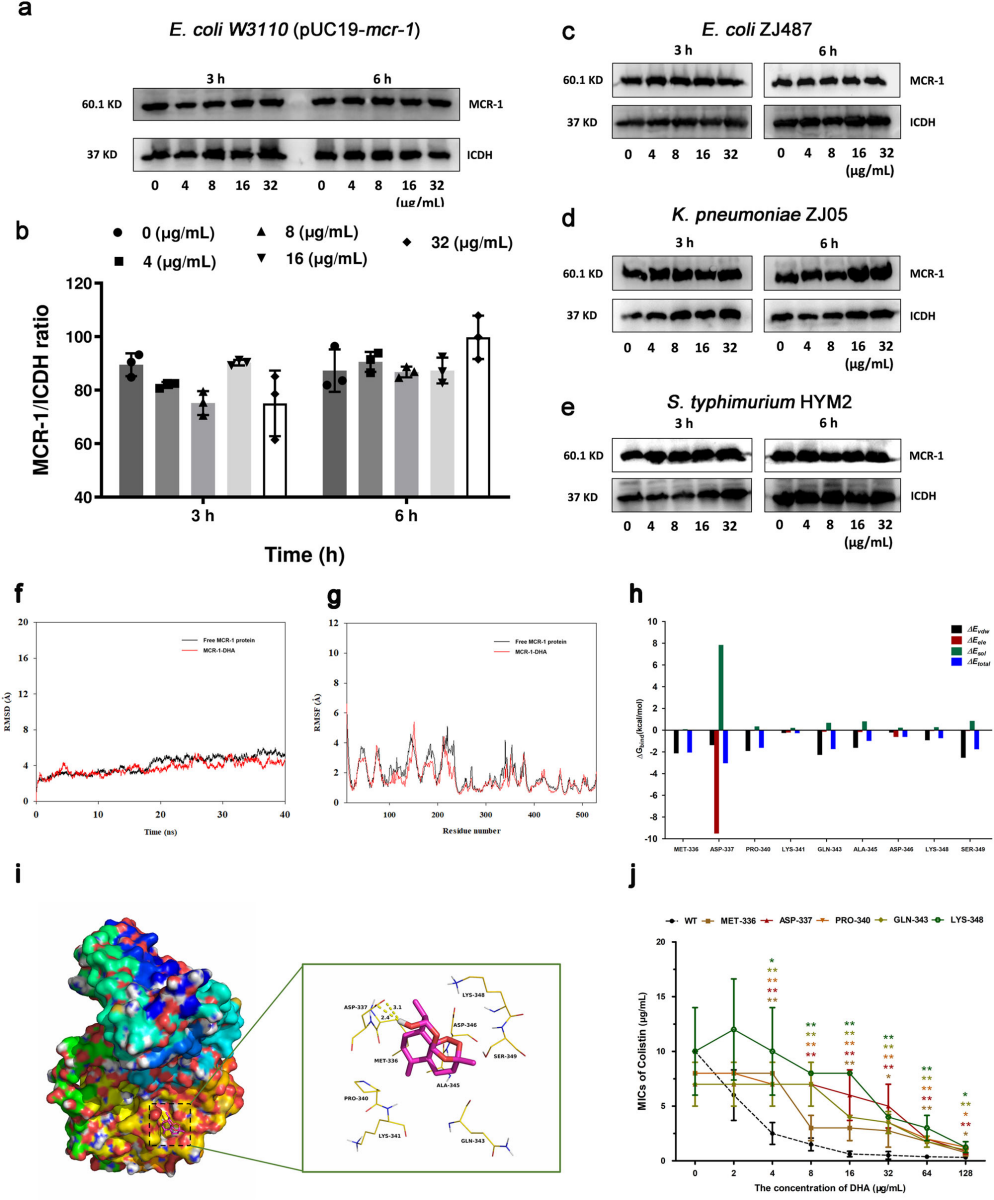
Direct engagement by DHA resulted in the inhibition of MCR-1 activity. **a**–**e** MCR-1 production in *E. coli* W3110 (pUC19-*mcr-1*) (**a**, **b**), *E. coli* ZJ487 (**c**), *K. pneumoniae* ZJ05 (**d**) and *S. typhimurium* HYM2 (**e**) cocultured with the indicated DHA treatment was determined by western blotting assays. **f** The root-mean-square deviations (RMSDs) of all the atoms of MCR-1-DHA complex with respect to its initial structure as a function of time. **g** RMSF of residues of the whole protein in the MCR-1-DHA complex and free MCR-1 during the 40 ns simulation. **h** Decomposition of the binding energy on a per-residue basis in the MCR-1-dihydroartemisinin complex. **i** The predicted binding mode of DHA in the MCR-1 binding pocket obtained from MD simulation. **j** The synergistic activity of DHA (at concentrations ≥4 µg/mL) with colistin for the bacteria harboring MCR-1 mutants was significantly decreased compared with *E. coli* W3110 (pUC19-*mcr-1*) (*n* = 4). *P < 0.05. **P < 0.01.

Results from the western blot assay showed that the expression of MCR-1 in the MCR-1-positive laboratory bacterial strain *E. coli* W3110 (pUC19-*mcr-1*) or *E. coli* ZJ487, *K. pneumoniae* ZJ05 and *S. typhimurium* HYM2 was not visibly affected by DHA treatment for 3 and 6 h (Fig. 6a–e), suggesting that DHA may inhibit MCR activity by direct engagement.

To explore the potential binding mode between DHA and MCR-1, molecular docking and molecular dynamics simulations were performed. As shown in Fig. 6f, the protein structures of the two systems were stabilized during the 40-ns simulation. The RMSF of these residues are shown in Fig. 6g, clearly depicting different flexibilities in the binding site of MCR-1 in the presence and absence of DHA. The majority of the residues in the MCR-1 binding site that bind with DHA showed a small degree of flexibility with a RMSF of <3 Å when compared with the free MCR-1, indicating that these residues seem to be more rigid as a result of binding to DHA.

In the MCR-1-DHA complex, the residue ASP-337 has a strong electrostatic (∆*E*_*ele*_) contribution, with a value of <−9.0 kcal/mol (Fig. 6h). Detailed analysis showed that residue ASP-337 is oriented to the hydroxyl group of DHA, leading to two hydrogen bond (bond lengths: 2.4 and 3.1 Å) interactions between the MCR-1 and DHA (Fig. 6i). In addition, the residue SER-349 with a ∆*E*_vdw_ of <−2.5 kcal/mol (Fig. 6h), has appreciable Van der Waals interactions with DHA because of the close proximity between the residues and DHA (Fig. 6i). Except for residues ASP-337 and SER-349, the majority of the decomposed energy interactions originated from Van der Waals interactions, apparently through hydrophobic interactions (i.e., MET-336 and PRO-340).

The total binding free energy for the MCR-1-DHA complex was calculated according to the MMGBSA approach, and an estimated ∆G_bind_ of −13.6 kcal/mol was found for DHA, suggesting that DHA can strongly bind to and interact with the binding site of MCR-1. In addition, the synergistic effects of DHA with colistin against *E. coli* W3110 harboring the MCR-1 mutants were significantly reduced at concentrations of 4 μg/mL or higher (Fig. 6j). These findings indicated that the results generated by the MD simulation on the MCR-1-DHA complex are reliable, which provided valuable information for further development of the MCR-1 inhibitors.

### DHA reduces the virulence of bacteria by hindering flagellar assembly and energy metabolism

**Fig. 7:**
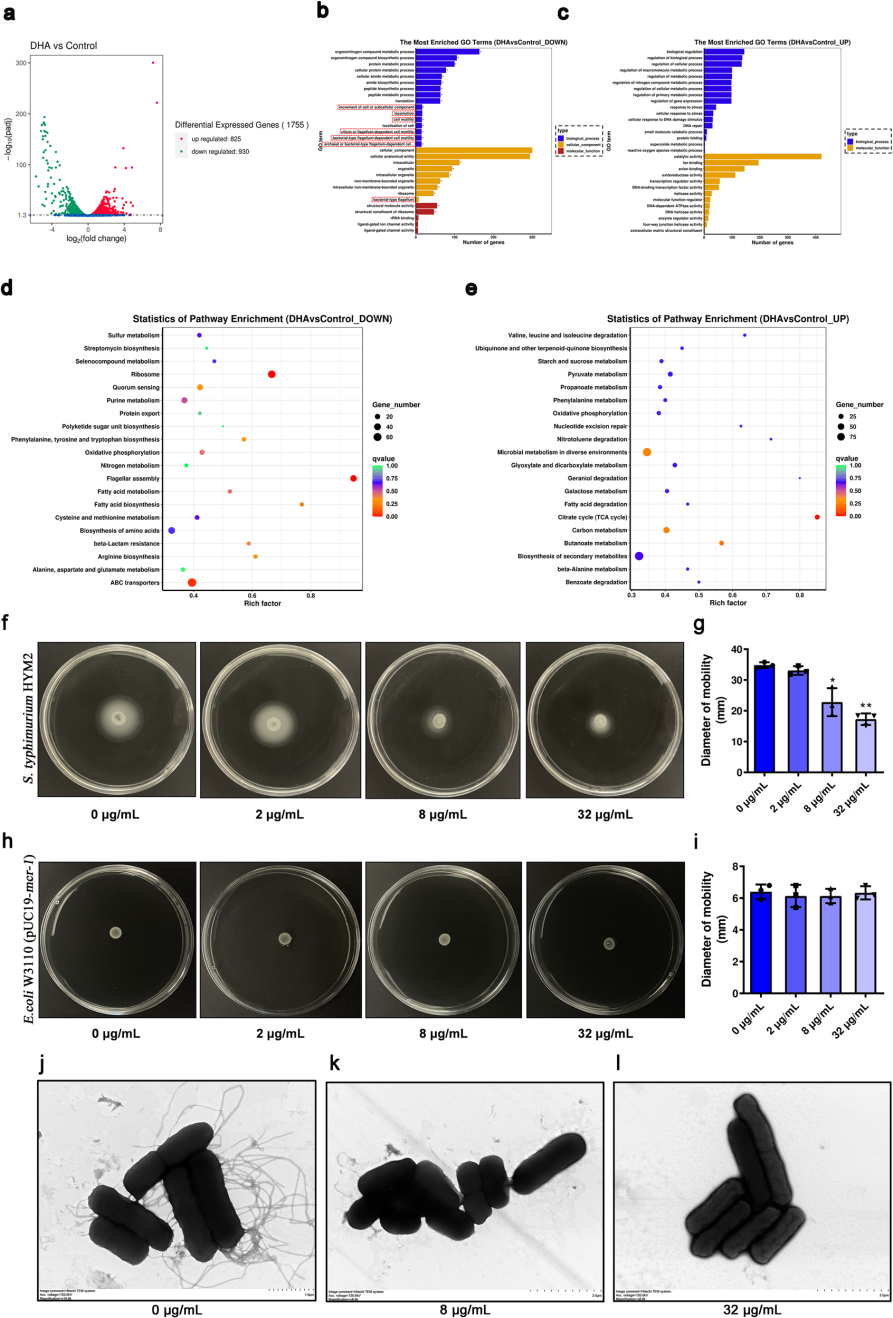
DHA reduces the virulence of bacteria by hindering the flagellar assembly and energy metabolism. **a** The differentially expressed genes of *E. coli* W3110 (pUC19-*mcr-1*) treated with DHA or control. **b**, **c** The most enriched GO terms associated with biological process, cellular component and molecular function, and the downregulated genes were mainly focused on the motility of bacteria (outlined in red). **d**–**i** The downregulated genes (**d**) and up-regulated genes (**e**) with DHA treatment compared with the control are shown. The motility of *S. typhimurium* HYM2 (**f**, **g**) and *E. coli* W3110 (pUC19-*mcr-1*) (**h**, **i**) on agar plates was photographed and the mobility diameter was measured (*n* = 3). **j**–**l** The flagellar morphology of *S. typhimurium* HYM2 treated with DHA at concentrations of 0 µg/mL (j), 8 µg/mL (**k**) and 32 µg/mL (**l**) was observed by TEM (Hitachi TEM system, Acc.voltage = 120.0 kV, bar = 1 μm (**j**), 2 μm (**k**, **l**)) (*n* = 3). *P < 0.05. **P < 0.01.

Transcriptome analysis can validate possible mechanisms of DHA in bacteria and screen for unknown mechanisms. The results of the transcriptome analysis revealed that 825 expressed genes and 930 expressed genes were upregulated and downregulated following 32 μg/mL DHA treatment which was associated with bacteria of biological processes, cellular components and molecular functions (Fig. 7a–c). As expected, some down-regulated genes were associated with flagellum-mediated bacterial motility (Fig. 7b). Similarly, statistics of pathway enrichment analysis further showed that the differentially expressed genes were mainly involved in flagellar assembly (down) and the tricarboxylic acid (TCA) cycle (up) (Fig. 7d, e). We first tested whether DHA influenced the motility of the tested bacteria. As shown in Fig. 7f, g, Supplementary Fig. 6b and 6c, the motility of the strains *S. typhimurium* HYM2 and *S. typhimurium* SL1344 was impaired when treated with different concentrations of DHA. Frequent pausing is common phenomena in *E. coli* flagellar growth, but not in *S. enterica*. And insufficient cytoplasmic flagellin supply leads to the pauses in flagellar growth in *E. coli*^19^. Maybe due to the insufficient cytoplasmic flagellin supply, flagellar-mediated movement was not observed in *E. coli* W3110 (pUC19-*mcr-1*) (Fig. 7h, i), Furthermore, DHA significantly inhibited the appearance of flagella of *S. typhimurium* HYM2 as visually observed by TEM, which was consistent with the SEM observation results (Fig. 7j, l).

**Fig. 8:**
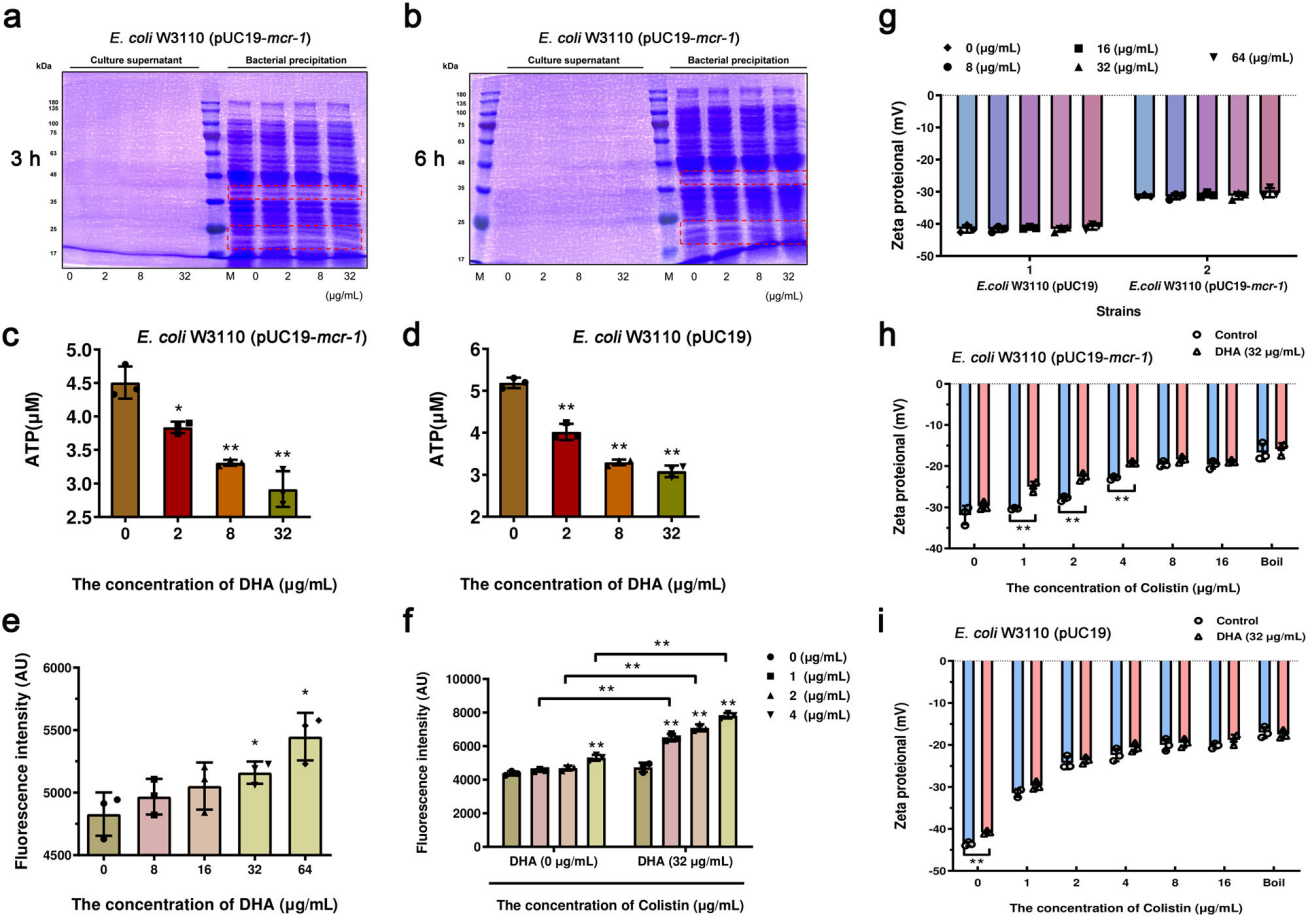
DHA increases the membrane damage capacity of colistin. **a**, **b** DHA addition decreased the expression of unknown proteins (outlined in red) in the bacterial precipitation of *E. coli* W3110 (pUC19-*mcr-1*) at 3 and 6 h. Samples were loaded on SDS–PAGE gels and analyzed by staining with Coomassie brilliant blue. **c**, **d** Detection of intracellular ATP levels in *E. coli* W3110 (pUC19-*mcr-1*) and *E. coli* W3110 (pUC19). **e**, **f** The permeability of the inner membrane of *E. coli* W3110 (pUC19-*mcr-1*) treated with DHA (0, 8–64 μg/mL), colistin (0, 1–4 μg/mL) or colistin (0, 1–4 μg/mL) in combination with DHA (32 μg/mL) for 30 min, probed with 5 μM PI. **g**–**i** Zeta potential of *E. coli* W3110 (pUC19-*mcr-1*) and *E. coli* W3110 (pUC19) treated with DHA (0, 8-64 μg/mL), colistin (0, 1–16 μg/mL) or colistin (0, 1–4 μg/mL) in combination with DHA (32 μg/mL) were performed. The experiments were repeated three times independently. *P < 0.05. **P < 0.01.

Second, the transcriptome analysis also indicated that DHA influenced energy metabolism (The expressed genes for TCA cycle were up-regulated), and thus, the situation of protein expression, ATP content and membrane damage of *E. coli* W3110 (pUC19-*mcr-1*) were detected. As shown in Fig. 8a, b, the expression of some small molecular weight proteins was inhibited in a dose-dependent manner. The acceleration of the TCA cycle promoted the accumulation of reactive oxygen species (ROS), and the accumulation of ROS often accompanied by a corresponding reduction in intracellular ATP levels in intracellular^20^. As shown in Fig. 8c, d, DHA treatment significantly inhibited intracellular ATP generation in *E. coli* W3110 (pUC19-*mcr-1*) and *E. coli* W3110 (pUC19) bacteria. Meanwhile, we used the fluorescent probes propidium iodide (PI) and zeta proteional to further estimate whether DHA affected bacterial membrane permeability and membrane electronegativity to enhance the antibacterial effect of colistin. DHA almost did not impact the fluorescence intensity of *E. coli* W3110 (pUC19-*mcr-1*), and did not affect the zeta proteional of *E. coli* W3110 (pUC19-*mcr-1*) and *E. coli* W3110 (pUC19) (Fig. 8e, g). However, the ability of polymyxin to damage the bacterial membrane was enhanced by DHA (32 μg/mL)(Fig. 8f, h, i). Taken together, our results established that DHA not only inhibits the activity of MCR-1, but also inhibits the flagellar assembly or the energy metabolism of Gram-negative bacteria.

### Artemisinin derivatives in combination with colistin protected mice from different Gram-negative bacterial infection models

**Fig. 9:**
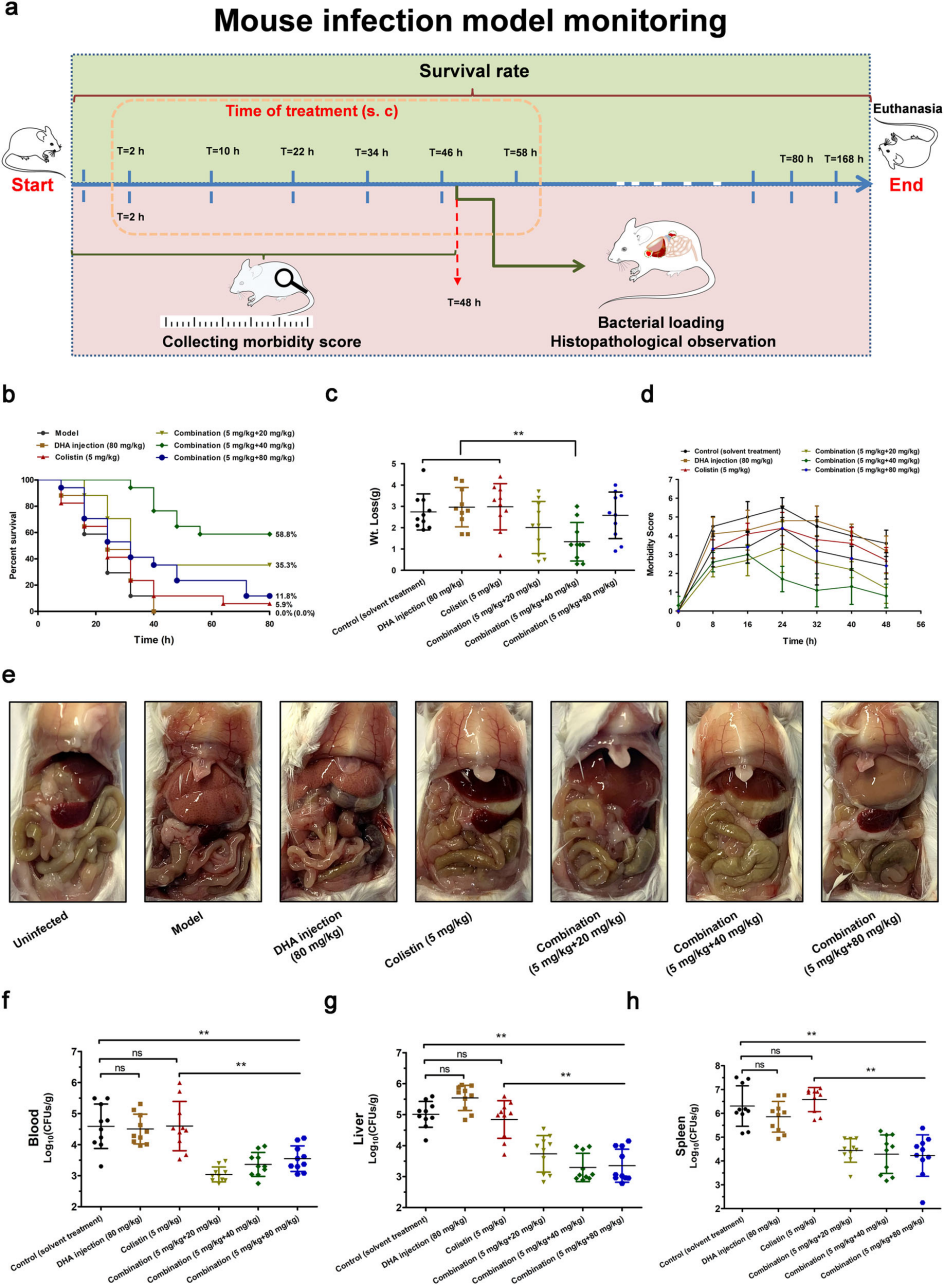
Effects of DHA injection and colistin combination therapy against *E. coli* W3110 (pUC19-*mcr-1*) in vivo. **a** Mouse infection model monitoring including survival rate, time of treatment, Wt. Loss (weight loss), morbidity score, bacterial loading and histopathological observation. **b** Survival plot of mice inoculated via intraperitoneal injection with *E. coli* W3110 (pUC19-*mcr-1*) (17 mice per group). **c**–**h** The Wt. Loss (**c**), morbidity score (**d**), histopathological observation (**e**) and the bacterial burden in the blood (**f**), liver (**g**) and spleen (**h**) were calculated (*n* = 10). **P < 0.01. ns indicates no significant.

The synergy of artemisinin derivatives in combination with colistin was further illustrated by different mouse models of Gram-negative bacterial infection. Firstly, a mouse model of systemic infection by *E. coli* W3110 (pUC19-*mcr-1*) was used to comprehensively analyze the synergy of DHA injection and colistin (Fig. 9a and Supplementary Fig. 6d).

As shown in Fig. 9b, administration of the low-dose combination (5 mg/kg + 20 mg/kg) and medium-dose combination (5 mg/kg + 40 mg/kg) improved the survival rate, with survival increasing from 0% (solvent-treated controls) to 35.3% and 58.8%, respectively. The mice treated with the medium-dose combination exhibited increased weight loss and morbidity under sub-lethal dose infection of *E. coli* W3110 (pUC19-*mcr-1*) (Fig. 9c, d). The medium-dose combination and low-dose combination treatment led to significant remission of pathological damage to organs in the abdomen, such as the liver and spleen (Fig. 9e and Supplementary Fig. 7). Notably, the high-doses of DHA injection had significant liver toxicity after multiple successive dosages. In addition, a reduction in CFU counts of more than one order of magnitude was observed in the blood, liver and spleen of the mice in all three combination therapy groups compared with the groups treated with DHA injection, colistin alone or the control (Fig. 9f–h).

**Fig. 10:**
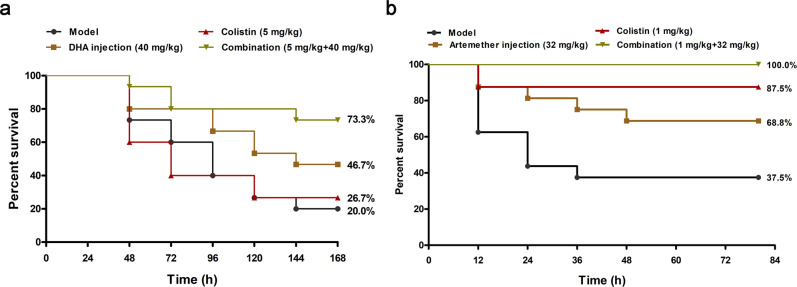
Effects of DHA injection/artemether injection monotherapy or combination therapy against *S. typhimurium* HYM2 and *A. baumannii* ATCC19606. **a**, **b** The survival rates of mice infected with *S. typhimurium* HYM2 (15 mice per group) (**a**) and *A. baumannii* ATCC19606 (16 mice per group) (**b**) were monitored for 7 days post-infection. The data represent the means and standard deviations from three separate experiments.

To further verify the generality of the synergistic effect of artemisinin derivatives in combination with colistin against different Gram-negative bacteria, such as *S. typhimurium* and *A. baumannii*. The mouse infection model by *S. typhimurium* HYM2 demonstrated that the survival rate of infection increased from 20.0% to 73.3% in the combination group (5 mg/kg + 40 mg/kg) compared to the control group (Fig. 10a). Interestingly, monotherapy with DHA injection also showed effective protection against infected mice, with a survival rate of 46.7%. In addition, for the *A. baumannii* ATCC19606 infection model (the synergistic effect of artemether and colistin on *A. baumannii* ATCC19606 (Supplementary Fig. 6f) was better than that on *A. baumannii* ATCC17978 (Supplementary Fig. 6g)), 100% of the mice treated with a combination of artemether injection (32 mg/kg) and colistin (1 mg/kg) survived until 80 h (Fig. 10b and Supplementary Fig. 6e).

Taken together, our results confirmed that DHA, at concentrations without affecting the growth of bacteria, could restore the bactericidal effect of colistin by simultaneously inhibiting the activity of MCR-1, affecting bacterial energy metabolism and impeding the assembly of bacterial flagella in vivo/in vitro.

## Discussion

The rise in antibiotic resistance in CRE coupled with weakness in developing new antibacterial drugs has urgently required the use of older polymyxins (mainly colistin and polymyxin B). Screening for potentiators from commercialized drugs (such as anti-parasitics and anti-virals) that effectively restore the antibacterial activity of polymyxins in vitro and in vivo is the most effective strategy^21^. Artemisinin derivatives are a class of anti-malarial drugs, and the anti-malarial mechanisms of artemisinin and its derivatives are as follows: a) artemisinin and its derivatives generate free radicals upon activation in vivo, followed by oxidative free radicals complexing with pivotal proteins in plasmodium to form covalent bonds, rendering the proteins non-functional leading to *Plasmodium* death; b) artemisinin and its derivatives selectively kill *Plasmodium* in the intraerythrocytic stage by affecting the function of the epithelium-mitochondria; and c) artemisinin and its derivatives are potent inhibitors of *Plasmodium* falciparum phosphatidylinositol-3-kinase (PfPI3K)^15,22^. These inhibitory effects may be similar in bacteria that inhibit flagellar assembly and energy metabolism such as TCA cycle, which are associated with both virulence and resistance.

Not all artemisinin derivatives have a significant synergistic antibacterial effect with colistin (Fig. 1b), and only after modification of specific structures can they exert excellent synergy, which would be an interesting study of research for artemisinin derivative chemical structure modification (Supplementary Fig. 1). In this sudy, we found that DHA in combination with polymyxin had a significant synergies on both *mcr-1* positive and negative bacteria, which means that DHA is not a specific MCR-1 inhibitor. The acceleration of the TCA cycle may lead to disturbances in intracellular ATP levels, bacterial energy metabolism, promoting the accumulation of reactive oxygen species (ROS), which may be the reason that DHA enhances energy metabolism the antibacterial activity of polymyxin against MCR-1-negative bacteria^20^. In addition, the screening strain *E. coli* W3110 (pUC19-*mcr-1*) carried the plasmid pUC19 containing ampicillin resistance. Therefore, we did not detect whether artemisinin derivatives have synergistic antibacterial effects with β-Lactam antibiotics. β-lactam antibiotics act on the bacterial wall, and thus there may also be good synergistic effects.

The preparation of artemisinin derivatives has the advantages of adequate dose basis for administration, significant clinical efficacy, a wide range of dosage forms and fewer poisonous-side effects^23^. To date, dihydroartemisinin tablets, artesunate tablets or injections and artemether injection have been used stably in clinic^23^. In future clinical settings, we can combine artemisinin derivative preparations with colistin or polymyxin B for rapid and precise treatment for different pan-drug resistance Gram-negative bacterial infectious diseases, such as *Enterobacter* pneumonia, bacterial gastroenteritis, nephropyelitis, urocystitis and bacteriaemia, to eliminate drug-resistant and sensitive clinical isolates in one fell swoop.

Critically, in human clinical chemotherapy, neither colistin nor polymyxin B is usually an option for monotherapy, mainly because the dose escalation that is required to achieve sufficiently high concentrations with the currently recommended dosing protocols risks the rapid onset of nephrotoxicity^24,25^. Therefore, by reducing the amount of polymyxins applied for clinical therapy, this combination may simultaneously retard the possibility of the spread of colistin resistance and decrease the risks of the onset of nephrotoxicity^26^.

In conclusion, our study shows that colistin in combination with commercialized artemisinin derivatives has immense potential as an alternative treatment option for combating infections caused by Gram-negative bacteria, especially MCR-positive CRE.

## Materials and methods

### Bacterial strains, cell lines, and chemicals

The bacteria including 14 MCR-1/3/5 producing bacterial strains and 11 MCR-negative bacterial strains, are listed in Supplementary Table 1 and are also described in our previous study^17,18,27^. *E. coli* W3110 (pUC19-*mcr-1*) carried a whole *mcr-1* gene originating from *K. pneumoniae* ZJ05. *E. coli* ATCC25922, *E. coli* W3110 (pUC19) (carrying a single plasmid of pUC19), *K. pneumoniae* ATCC700603, *P.aeruginosa* ATCC27853, *A. baumanii* ATCC19606, *A. baumanii* ATCC17978 and *S. typhimurium* ATCC14028 were used as negative quality control strains, which were purchased from the American Type Culture Collection (ATCC). The primers of MCR-1-WT and mutants are listed in Supplementary Table 2 Site-directed mutagenesis of *mcr-1* was performed using a QuikChange site-directed mutagenesis kit (Stratagene, La Jolla, CA, USA). The pUC19-*mcr*-*1* and mutants were transformed into *E. coli* W3110 (Genotype: F- lambda- IN(rrnD-rrnE)1 rph-1, BioVector NTCC Inc., Beijing, China) for minimum inhibitory concentration (MIC) determination.

J774 cells, Vero cells and HeLa cells in current study were obtained from ATCC. Our laboratory uses the gold standard STR method for cell identification and confirmed free from mycoplasma.

Artemisinin and its derivatives were purchased from the Chengdu Deruike Biotechnology Co., Ltd. (Chengdu, China) (Supplementary Fig 8). And the composition of DHA injection was listed in Supplementary Table 3 The artemether injection (Ratification No in China: GYZZ H10900011) was purchased from KPC Pharmaceuticals, Inc (Kunming, China). All of the antibiotics used in this study were bought from the China Institute of Veterinary Drug Control, CSNpharm, Inc., the National Institute for the Control of Pharmaceutical and Biological Products (Beijing, China) and the Dalian Meilun Biotechnology Co., Ltd. Unless otherwise stated, all chemicals used in this study were purchased from Sigma–Aldrich Inc. (St. Louis MO, USA).

### MIC determination

The synergistic antibacterial effect between artemisinin or its derivatives and colistin against MCR-1-positive Gram-negative bacterial strains and MCR-1-negative Gram-negative bacterial strains was identified by checkerboard MIC assays, and was carried out using the broth microdilution (BMD) method essentially as described in the Clinical and Laboratory Standards Institute (CLSI) protocol^28^. Briefly, each row of antibiotics was serially diluted twofold, and each column of compounds was serially diluted twofold. All MICs were determined in quadruplicate. The synergistic efficacies of antibiotics combined with compounds were assessed by calculating the fractional inhibitory concentration (FIC) index values. FIC index = (MIC of compounds alone/MIC of compounds in combination) + (MIC of antibiotics alone/MIC of antibiotics in combination). “FIC index > 4” was defined as antagonism, “0.5 < FIC index ≤ 4” was considered indifferent, and “FIC index ≤ 0.5” was defined as synergy^29^.

### Growth curves and bactericidal test

Overnight, four representative strains of MCR-1 positive bacteria, including *E. coli* W3110 (pUC19-*mcr-1*), *E. coli* ZJ487, *K. pneumoniae* ZJ05 and *S. typhimurium* HYM2, were cultured at 37 °C to obtain an OD_600 nm_ value of 0.3. The cultures were divided into five 50 mL sterilized Erlenmeyer flasks, and DHA was added to the cultures at final concentrations of 0, 16, 32, 64, and 128 μg/mL. The bacteria were cultured for up to 6–7 h, and bacterial growth was detected by measuring the OD_600 nm_ every 30 min.

The bactericidal effect of DHA and colistin was evaluated by time-killing assays and plating sterilization tests. In brief, mid-logarithmic-phase bacteria of *E. coli* W3110 (pUC19-*mcr-1*), *E. coli* ZJ487, *K. pneumoniae* ZJ05 and *S. typhimurium* HYM2 were separately diluted to 5 × 10^5^ CFU/mL in 96-well plates with LB broth, and colistin (1/8 × MIC), DHA (1/8 × MIC) and colistin (1/8 × MIC) combined with DHA (1/8 ×  MIC) were added. The bacterial cultures in 96-well plates were incubated at 37 °C and were removed at 0–24 h post-inoculation for bacterial counts. Ten microliters of serial 10-fold dilutions of the bacterial cultures were spread onto Luria-Bertani (LB) agar plates. Bacterial colonies were counted after incubation at 37 °C overnight. In addition, to clarify the sterilization level of the combination, 5 μL of serial 10-fold dilutions (1 × 10^8^ CFU/mL, 1 × 10^7^ CFU/mL, 1 × 10^6^ CFU/mL and 1 × 10^5^ CFU/mL) of bacterial cultures of *E. coli* W3110 (pUC19-*mcr-1*) with different treatments were spread onto LB agar at 0, 3, and 12 h to obtain the number of colonies after an incubation of 24 h at 37 °C.

### Live/dead bacteria staining

Live/dead bacteria staining was used to more visually observe the live or dead status of the tested bacteria. Mid-logarithmic-phase bacteria of *E. coli* W3110 (pUC19-*mcr-1*) and *S. typhimurium* HYM2 were diluted in LB broth, and DHA (32 μg/mL), colistin (1/0.5 μg/mL) or the combination was added for 5 h at 37 °C. Then, the culture was centrifuged at 5000 rpm for 10 min to collect bacteria, which were further resuspended in sterile PBS buffer to obtain an OD_600nm_ of 0.5. The live bacteria were labeled green fluorescently and dead bacteria were labeled red fluorescently using the LIVE/DEAD BacLight Bacterial Viability Kit (Cat. No. L7012, Thermo Fisher Scientific, (China), Co., Ltd) under the guidance of the manufacturer’s instructions.

### Scanning electron microscope (SEM) assays and transmission electron microscopy (TEM) analysis

The tested bacteria of *E. coli* W3110 (pUC19-*mcr-1*) and *S. typhimurium* HYM2 were treated with DHA (32 μg/mL), colistin (0.5/1 μg/mL) or 32 μg/mL DHA plus 0.5/1 μg/mL colistin for 5 h, respectively, washed with PBS and resuspended in PBS to obtain an OD_600nm_ of 0.5. Then, the samples were incubated with polylysine (0.1%), washed with PBS and fixed in glutaraldehyde overnight at 4 °C. Finally, the morphological changes of the tested bacteria were observed by SEM.

For TEM analysis, the colony of *S. typhimurium* HYM2 was fixed with 50 μL sterile glutaraldehyde phosphate buffer. A 10 μL sample was split on copper TEM grids and incubated for 10 min at room temperature. Bacterial samples were stained with uranyl acetate/lead citrate and observed using a transmission electron microscope (Hitachi Co, Ltd, Japan).

### Serial passage colistin-resistance test

A serial passage colistin-resistance test was used to determine whether the synergy of DHA and colistin is tenacious for different bacteria with or without MCR-1 under pressure for antibiotic selection. The overnight culture tested bacteria were adjusted to obtain an OD_600nm_ of 0.1, and colistin (1/8 × MIC), DHA (1/8 × MIC), combination (1/8 × MIC of colistin under 32 μg/mL of DHA) or solvent control was added to the culture for 24 h at 37 °C with shaking. Then, the different treated bacteria were adjusted to obtain an OD_600nm_ of 0.1 and the same processing as the previous day was added until 30 days. The MIC of colistin for all the bacteria in each group was detected per 3 days until 30 days.

### Invasion assay and immunofluorescence microscopy

The invasion ability of *E. coli* in Vero cells and *S. typhimurium* in HeLa cells was detected using a verified gentamicin protection assay^30,31^. In brief, 5 × 10^5^ Vero cells or HeLa cells were plated into 24‐well plates and cultured overnight. The cells in wells with different treatments (32 μg/mL DHA, 0.25/1/2 μg/mL colistin, combination and control) were infected by *E. coli* or *S. typhimurium* at a MOI of 100 and incubated at 37 °C for 2, 4 and 6 h. Then, the cells were washed, and 100 μg/mL gentamicin was added to each well to clear away uninvaded bacteria. Then, the cells were permeabilized and the bacteria in cells were counted by plating on LB agar plates and incubated at 37 °C overnight.

For immunofluorescence microscopy, HeLa cells in the wells were infected with *S. typhimurium*, which was supplemented with different concentrations of DHA (0, 2, 8, and 32 μg/mL) and incubated at 37 °C for 2 h. After treatment with gentamicin, cells were fixed with 4% paraformaldehyde for 30 min and blocked with 5% BSA for 1 h. The cells were orderly incubated with an anti‐*S. typhimurium* rabbit antibody (1:2000) for 1 h, incubated with the secondary antibody (1:1000, goat anti‐rabbit conjugated‐IgG/Alexa Fluor 488) for 30 min, permeabilized with 0.3% Triton X‐100 for 5 min, incubated with anti‐*S. typhimurium* rabbit antibody (1:2000) for 1, incubated with the secondary antibody (1:1000, goat anti‐rabbit conjugated‐IgG /Alexa Fluor 594) for 30 minutes. The cells were washed three times with PBS buffer after each incubation with antibody. The HeLa cell nuclei were stained with DAPI (4′,6-diamidino-2-phenylindole), and the immunofluorescence was observed and photographed using an Olympus fluorescence microscope.

### Safety evaluation

The potential toxicity of DHA to the J774 cells, Vero cells, HeLa cells and mouse peritoneal macrophages was determined using a Cytotoxicity Detection Kit (LDH) (Roche, Basel, Switzerland) at an absorbance of 490 nm after coincubation for 6 h. Cells in a well treated with Triton X-10 (0.2%) were used as a positive control, and untreated cells in a well served as a negative control.

### Western blot assays

After treatment with DHA (0, 4-32 μg/mL) for 3 or 6 h, bacterial cultures were collected to prepare western blot assays sample. The membrane was blocked, and incubated with primary antibody against MCR-1 (prepared from mouse) and goat anti-mouse IgG secondary antibody (HRP). Finally, the membrane was visualized with an enhanced chemiluminescence substrate and the blots were quantified using ImageJ software (GE Healthcare Life Sciences, UK). Moreover, the samples of culture supernatant and bacterial precipitation from *E. coli* W3110 (pUC19-*mcr-1*) were separated by SDS–PAGE and stained with Coomassie brilliant blue.

### Molecular docking and molecular dynamics (MD) simulations

A molecular docking study was performed to investigate the binding mode between dihydroartemisinin and MCR-1 using AutoDock Vina 1.1.2^32^. The 3D structure of the dihydroartemisinin was drawn by ChemBioDraw Ultra 14.0 and ChemBio3D Ultra 14.0 software. The AutoDockTools 1.5.6 package was employed to generate the docking input files^33,34^. For Vina docking, the default parameters were used not mentioned. Then an MD study was performed to revise the docking result.

The Amber 14 and AmberTools 15 programs were used for MD simulations of the selected docked pose^35^. Amentoflavone was first prepared by ACPYPE, a tool based on ANTECHAMBER for generating automatic topologies and parameters in different formats for different molecular mechanics programs, including calculation of partial charges^36^. Then, the forcefield “leaprc.gaff” (generalized amber forcefield) was used to prepare the ligand, while “leaprc.ff14SB” was used for the receptor. Equilibration of the solvated complex was performed by carrying out a short minimization (2000 steps of each steepest descent and conjugate gradient method), 1000 ps of heating, and 500 ps of density equilibration with weak restraints using the GPU accelerated PMEMD (Particle Mesh Ewald Molecular Dynamics) module. At last, 40 ns of MD simulations were carried out. All molecular dynamics were performed on a Dell Precision T5500 workstation.

The binding free energies (ΔGbind in kcal/mol) were calculated using the Molecular Mechanics/Generalized Born Surface Area (MM/GBSA) method, implemented in AmberTools 15. Moreover, to identify the key protein residues responsible for the ligand binding process, the binding free energy was decomposed on a per-residue basis. For each complex, the binding free energy of MM/GBSA was estimated as ΔGbind= Gcomplex‒Gprotein‒Gligand, where ΔGbind is the binding free energy and Gcomplex, and Gprotein and Gligand are the free energies of the complex, protein, and ligand, respectively.

### RNA isolation and transcriptome analysis

Overnight cultured *E. coli* W3110 (pUC19-*mcr-1*) was diluted in fresh LB broth with or without DHA (32 µg/mL) and cultured for 4–5 h to the exponential phase at OD_600nm_ = 1.0. The bacterial cells were harvested, and total RNA was extracted according to the protocol of the manufacturer. The construction and sequencing of cDNA libraries and transcriptome data collection were performed by the Allwegene BioTech Co.,Ltd. (Beijing, China).

### Surface motility of *S. typhimurium* and *E. coli*

Overnight cultures of *S. typhimurium* HYM2, *S. typhimurium* SL1344 or *E. coli* W3110 (pUC19-*mcr-1*) were diluted with LB broth to obtain an OD_600nm_ of 0.5. Three microliters of bacterial suspension was dropped on 0.3% agar plates containing different concentrations of DHA (0, 2-32 µg/mL). After incubation at 37 °C for 12-16 h, the motility of *S. typhimurium* HYM2, *S. typhimurium* SL1344 and *E. coli* W3110 (pUC19-*mcr-1*) on plates were photographed and the mobility diameter was measured.

### Estimation of the zeta potential

One hundred microliters of overnight-cultured bacterial strains *E. coli* W3110 (pUC19-*mcr-1*) or *E. coli* W3110 (pUC19) was inoculated in 10 mL of fresh LB broth at 37 °C for 2 h and cultured to the logarithmic growth phase. The bacteria were centrifuged at 12000 rpm for 10 min to collect bacterial cells, washed and resuspended in 0.5 mM potassium phosphate buffer (pH = 7.4) to obtain a dispersion system of OD_600nm_ = 0.5. Then, the bacterial cells were supplemented with different concentrations of DHA (0 µg/mL, 8–64 µg/mL) or different concentrations of colistin (0, 1–16 µg/mL) with or without DHA (32 µg/mL) and statically cultured at 37 °C for 2 h. Boiled bacterial cells (100 °C) were used as a positive control. The zeta potential was detected by a Nano ZS (Malvern, the United Kingdom) Zeta sizer in a disposable zeta cell.

### Intracellular ATP determination

Overnight-cultured bacterial strains *E. coli* W3110 (pUC19-*mcr-1*) or *E. coli* W3110 (pUC19) were 1:100 inoculated in LB medium and added various concentrations of DHA (0, 2–32 µg/mL) at 37 °C for 5 h, respectively. Bacterial cells were collected, washed and resuspended in PBS buffer. Then, the intracellular ATP levels were detected according to the manufacturer’s protocol and calculated based on the standard curve.

### Inner membrane (IM) permeability determination

One hundred fifty microliters of overnight-cultured *E. coli* W3110(pUC19-*mcr-1*) cells was mixed with 50 μL of DHA (0, 8–64 μg/mL) or colistin (0, 1–4 μg/mL) with or without DHA (32 μg/ml) and incubated at 37 °C for 30 min. Then, 5 μM propidium iodide (PI) was added for coincubation at 37 °C for 30 min, and the fluorescence intensity was measured at an excitation wavelength of 535 nm and emission wavelength of 617 nm using an Infinite M200 microplate reader (TECAN, Switzerland).

### Mouse infection model assays

Six- to eight-week-old female BALB/c mice (20 ± 2 g) were obtained from the Liaoning Changsheng Biotechnology Co., Ltd. Animal experiments were approved by the Animal Care and Use Committee of Jilin University.

The formal experiment was preceded by an effective dose screening of colistin (2.5, 5, and 10 mg/kg for *E. coli* W3110 (pUC19-*mcr-1*); 0.5, 1, and 2 mg/kg for *A. baumannii* ATCC19606) (*n* = 10, per group). The mice (*n* = 17, per group) were intraperitoneally infected with *E. coli* W3110 (pUC19-*mcr-1*) (1 × 10^9^ CFU with 5% mucin (M1778; Sigma–Aldrich)) to cause a systemic infection. After infection for 2 h, the mice were subcutaneously administered with colistin (5 mg/kg), DHA injection (80 mg/kg), colistin (5 mg/kg) in combination with DHA injection (20 mg/kg, 40 mg/kg or 80 mg/kg) or control solvent every 12 h (the first time was 8 h apart). The number of live mice was recorded every 8 h until 80 h post-infection.

The mouse (*n* = 16, per group) intraperitoneal infection model *A. baumannii* ATCC19606 (1 × 10^8^ CFU with 5% mucin (M1778; Sigma–Aldrich)) was also used to confirm the synergistic effect of colistin (1 mg/kg) and artemether injection (strength: 1 mL:80 mg)^37^. The mice were subcutaneously administered with colistin (1 mg/kg), artemether injection (32 mg/kg), colistin (1 mg/kg) in combination with artemether injection (32 mg/kg) or control solvent every 12 h (the first time was 8 h apart).

For *S. typhimurium* infection analysis, the mice (*n* = 15, per group) were housed for 5 days with water containing streptomycin (5 mg/mL) before infection and were orally gavaged with 5 × 10^7^ CFU of *S. typhimurium* HYM2. After infection for 2 h, the mice were orally gavaged with colistin (5 mg/kg), DHA injection (40 mg/kg), colistin (5 mg/kg) in combination with DHA injection (40 mg/kg) or control solvent every 12 h.

For bacterial loading analysis, weight analysis, and morbidity assays, the mice (*n* = 10, per group) were intraperitoneally infected with 5 × 10^8^ CFU of *E. coli* W3110 (pUC19-*mcr-1*) and treated as described above. The morbidity scores were judged and counted following the approved method^38^. Then, the mice were weighed and euthanized for pathological observation to obtain the bacterial burden in blood, livers, and spleens.

### Ethics

Animal experiments were approved by the Animal Care and Use Committee of Jilin University (No. ALKT202102003) and were operated following the guidelines of this committee. All the participators signed written informed consents.

### Statistics and reproducibility

Statistical analysis was performed using SPSS version 19.0 and GraphPad Prism 7.0, and the data are presented as the mean ± standard deviation (SD). Student’s *t*-test was used to determined the significance between the two groups. and differences were considered statistically significant when *P*-values were <0.05. The sample sizes and number of replicates and how replicates were defined in figure legend.

### Reporting summary

Further information on experimental design is available in the Nature Research Reporting Summary linked to this paper.

## Supplementary information

Supplementary information (PDF)

Description of Additional Supplementary Files

Supplementary Data 1

Reporting summary

## Data Availability

Source data underlying Figs. 1–2, 4–5, 7–10 and Supplementary Fig 6 are in the Supplementary Data file. Supplementary Fig 9 is the uncropped western blotting images. The accession number of transcriptome data are PRJNA866889. And all other data included in this study are available upon request by contact with the corresponding author.

## References

[1] Wang Y (2017). Comprehensive resistome analysis reveals the prevalence of NDM and MCR-1 in Chinese poultry production. Nat. Microbiol..

[2] Liu YY (2015). Emergence of plasmid-mediated colistin resistance mechanism MCR-1 in animals and human beings in China: a microbiological and molecular biological study. Lancet.

[3] Hameed F (2019). Plasmid-mediated mcr-1 gene in *Acinetobacter baumannii* and *Pseudomonas aeruginosa*: first report from Pakistan. Rev. da Soc. Brasileira de. Med. Tropical..

[4] Xu Y (2018). An evolutionarily conserved mechanism for intrinsic and transferable polymyxin resistance. Mbio.

[5] Liu X (2022). Emergence of colistin-resistant hypervirulent Klebsiella pneumoniae (CoR-HvKp) in China. Emerg. Microbes Infect..

[6] Pituch H (2002). Variable flagella expression among clonal toxin A-/B+Clostridium difficile strains with highly homogeneous flagellin genes. Clin. Microbiol. Infect..

[7] Luo G (2016). flrA, flrB and flrC regulate adhesion by controlling the expression of critical virulence genes in *Vibrio alginolyticus*. Emerg. Microbes Infect..

[8] Green, C. A. et al. Engineering the flagellar type III secretion system: improving capacity for secretion of recombinant protein. *Microbial Cell Factories* **18**, 10 (2019). 10.1186/s12934-019-1058-4 PMC6337784 30657054

[9] Tu Y (2016). Artemisinin-a gift from traditional Chinese medicine to the world (Nobel Lecture). Angew. Chem..

[10] Gosling RD, Okell L, Mosha J, Chandramohan D (2011). The role of antimalarial treatment in the elimination of malaria. Clin. Microbiol Infect..

[11] Tschan S, Kremsner PG, Mordmüller B (2012). Emerging drugs for malaria. Expert Opin. Emerg. Drugs.

[12] Li G. Q. at al. Clinical trials of artemisinin and its derivatives in the treatment of malaria in China. *Transactions of the Royal Society of Tropical Medicine & Hygiene*. (Supplement_1), S5-6 (1994). 10.1016/0035-9203(94)90460-x 8053027

[13] Mithwani S (2004). Population pharmacokinetics of artemether and dihydroartemisinin following single intramuscular dosing of artemether in African children with severe falciparum malaria. Br. J. Clin. Pharmacol..

[14] Chen Y (2021). Dihydroartemisinin attenuated the symptoms of mice model of systemic lupus erythematosus by restoring the Treg/Th17 balance. Clin. Exp. Pharmacol. Physiol..

[15] Cheong D (2020). Anti-malarial drug, artemisinin and its derivatives for the treatment of respiratory diseases. Pharmacol. Res..

[16] Efferth, T. From ancient herb to versatile, modern drug: Artemisia annua and artemisinin for cancer therapy. *Semin. Cancer Biol.* S1044579X17300299 (2017). 10.1016/j.semcancer.2017.02.009 28254675

[17] Guo, Y. et al. Honokiol restores polymyxin susceptibility to MCR-1-positive pathogens both in vitro and in vivo. *Appl. Environ. Microbiol.* **86**, e02346 (2019). 10.1128/AEM.02346-19 PMC7028959 31862719

[18] Zhou Y (2019). Discovery of a potential MCR-1 inhibitor that reverses polymyxin activity against clinical mcr-1-positive *Enterobacteriaceae*. J. Infect..

[19] Zhao Z (2018). Flagella elongation revealed by single cell real-time fluorescence imaging. Nat. Commun..

[20] Liu Y (2020). Anti-HIV agent azidothymidine decreases Tet(X)-mediated bacterial resistance to tigecycline in *Escherichia coli*. Commun. Biol..

[21] Wang TZ, Kodiyanplakkal RPL, Calfee DP (2019). Antimicrobial resistance in nephrology. Nat. Rev. Nephrol..

[22] Vafa Homann M (2017). Detection of malaria parasites after treatment in travelers: a 12-months longitudinal study and statistical modelling analysis. EBioMedicine.

[23] Ansari MT (2013). Malaria and artemisinin derivatives:an updated review. Mini Rev. Med. Chem..

[24] Smith NM (2020). Using machine learning to optimize antibiotic combinations: dosing strategies for meropenem and polymyxin B against carbapenem-resistant *Acinetobacter baumannii*. Clin. Microbiol. Infect..

[25] Tsuji BT (2019). International Consensus Guidelines for the Optimal Use of the Polymyxins.: Endorsed by the American College of Clinical Pharmacy (ACCP), European Society of Clinical Microbiology and Infectious Diseases (ESCMID), Infectious Diseases Society of America (IDSA), International Society for Anti-infective Pharmacology (ISAP), Society of Critical Care Medicine (SCCM), and Society of Infectious Diseases Pharmacists (SIDP). Pharmacotherapy.

[26] Ahmed ES (2020). Colistin and its role in the Era of antibiotic resistance: an extended review (2000–2019). Emerg. Microbes Infect..

[27] Zhou Y (2018). Pterostilbene, a potential MCR-1 inhibitor that enhances the efficacy of polymyxin B. Antimicrobial Agents Chemother..

[28] CLSI, Performance standards for antimicrobial susceptibility testing. Nccls Document M S (2012).

[29] Odds FC (2003). Synergy, antagonism, and what the chequerboard puts between them. J. Antimicrob. Chemother..

[30] Liu Y (2020). Metformin restores tetracyclines susceptibility against multidrug resistant bacteria. Adv. Sci..

[31] Lv, Q. at al. Inhibition of the type III secretion system by syringaldehyde protects mice from Salmonella enterica serovar Typhimurium. *J. Cell. Mol. Med.* **23**, 4679-4688 (2019). 10.1111/jcmm.14354 PMC6584516 31066220

[32] Trott O, Olson AJ (2009). AutoDock Vina: improving the speed and accuracy of docking with a new scoring function, efficient optimization, and multithreading. J. Comput. Chem..

[33] Sanner MF (1998). Python: a programming language for software integration and development. J. Mol. Graph. Model..

[34] Morris GM (2009). AutoDock4 and AutoDockTools4: automated docking with selective receptor flexibility. J. Computational Chem..

[35] Salomon-Ferrer R (2013). Routine microsecond molecular dynamics simulations with AMBER on GPUs. 2. Explicit solvent particle mesh ewald. J. Chem. Theory Comput..

[36] Wang J (2006). Automatic atom type and bond type perception in molecular mechanical calculations. J. Mol. Graph. Model..

[37] Dudhani RV, Turnidge JD, Nation RL (2010). fAUC/MIC is the most predictive pharmacokinetic/pharmacodynamic index of colistin against Acinetobacter baumannii in murine thigh and lung infection models. J. Antimicrobial Chemother..

[38] Daly SM, Sturge CR, Felder-Scott CF, Geller BL, Greenberg DE (2017). mcr-1 inhibition with peptide-conjugated phosphorodiamidate morpholino oligomers restores sensitivity to polymyxin in *Escherichia coli*. Mbio.

