# Supplementary material for: Commercialized artemisinin derivatives combined with colistin protect against critical Gram-negative bacterial infection
Source: Commun Biol. 2022 Sep 8;5:931. doi: 10.1038/s42003-022-03898-5 (PMC9458715; doi:10.1038/s42003-022-03898-5)
Supplement: Supplementary file 1 — Supplementary information (PDF) [file 42003_2022_3898_MOESM1_ESM.pdf]

Supplementary Figure and Figure legends:

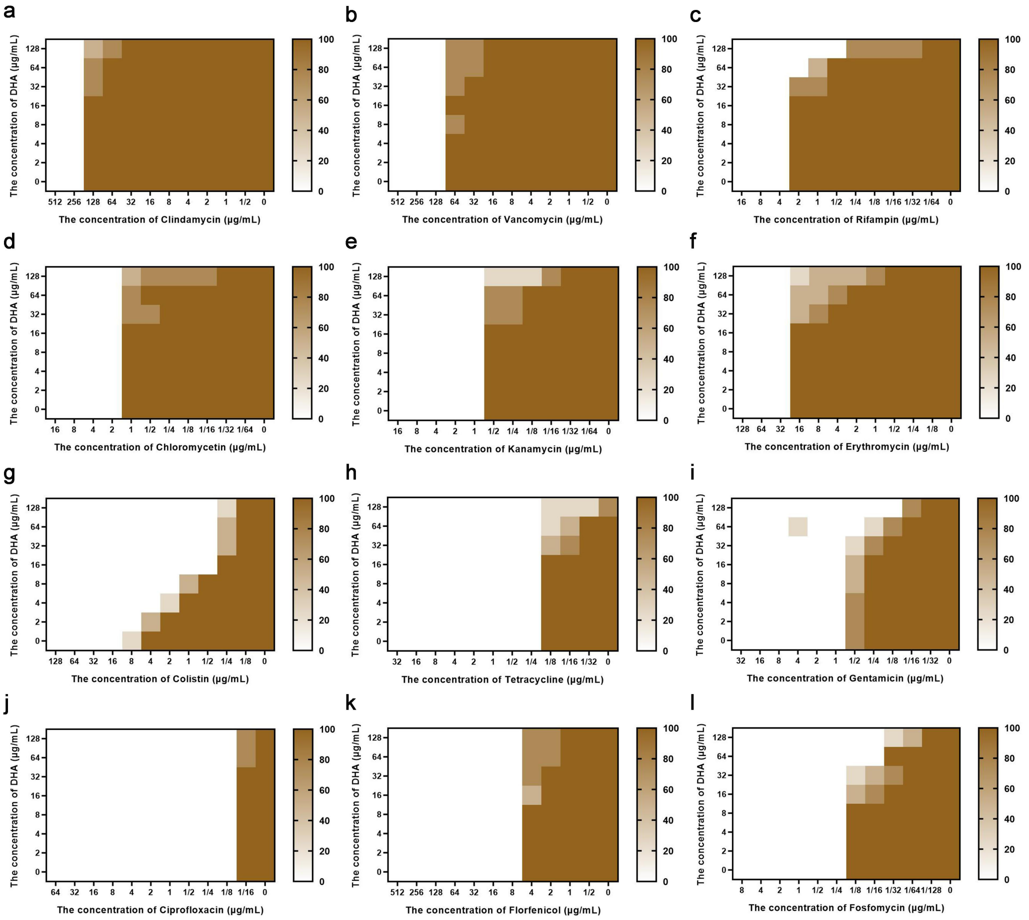

Supplementary Fig 1. The checkerboard MIC detection of DHA in combination with 12 different antibiotics against *E. coli* W3110 (pUC19-*mcr-1*). a-l The checkerboard MICs were determined in quadruplicate, and used the color shade to indicate the probability of bacterial growth in the well. The more bacterial growth visible to the naked eye, the darker the color.

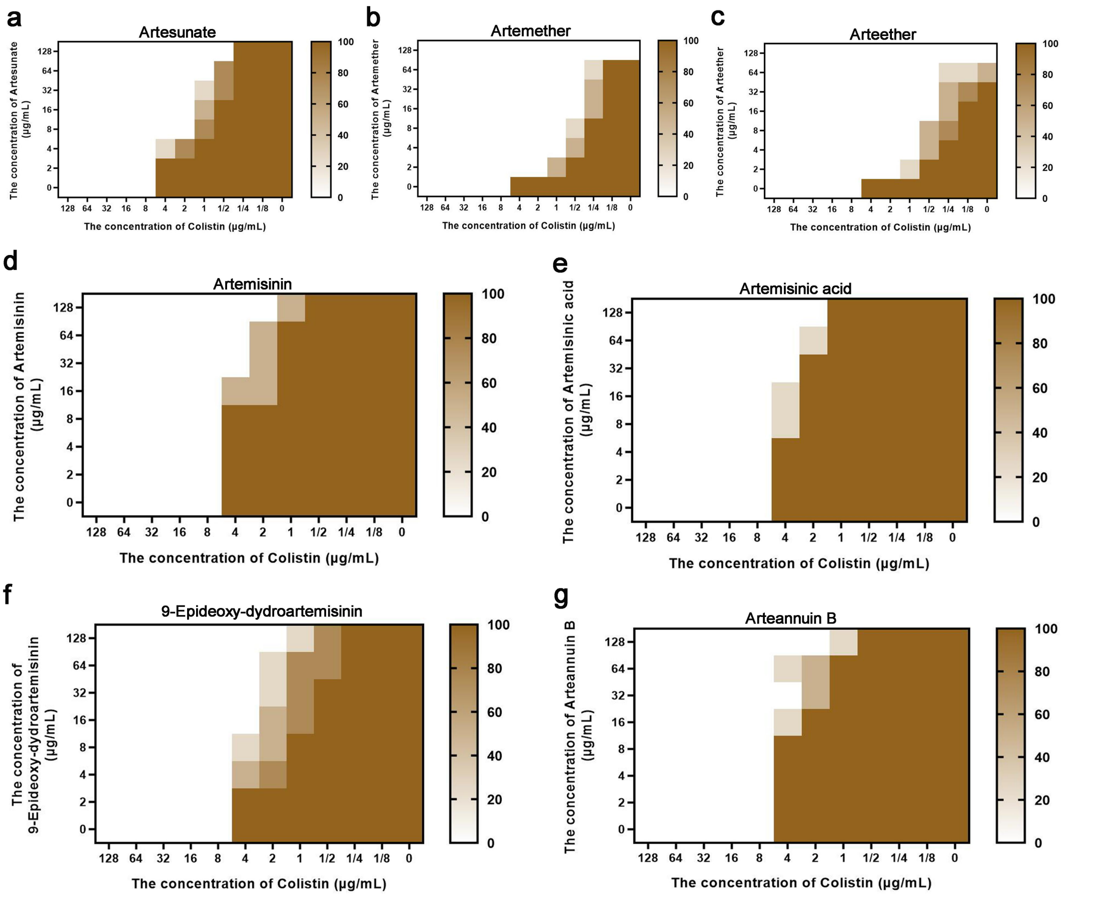

Supplementary Fig 2. The checkerboard MIC detection. a-g The checkerboard MIC detection of artemisinin derivatives, artesunate (**a**), artemether (**b**), arteether (**c**), artemisinin (**d**), artemisinic acid (**e**), 9-epideoxy-dydroartemisinin (**f**) and arteannuin B (**g**), in combination with colistin against *E. coli* W3110 (pUC19-*mcr-1*). The checkerboard MICs were determined in quadruplicate, and used the color shade to indicate the probability of bacterial growth in the well. The more bacterial growth visible to the naked eye, the darker the color.

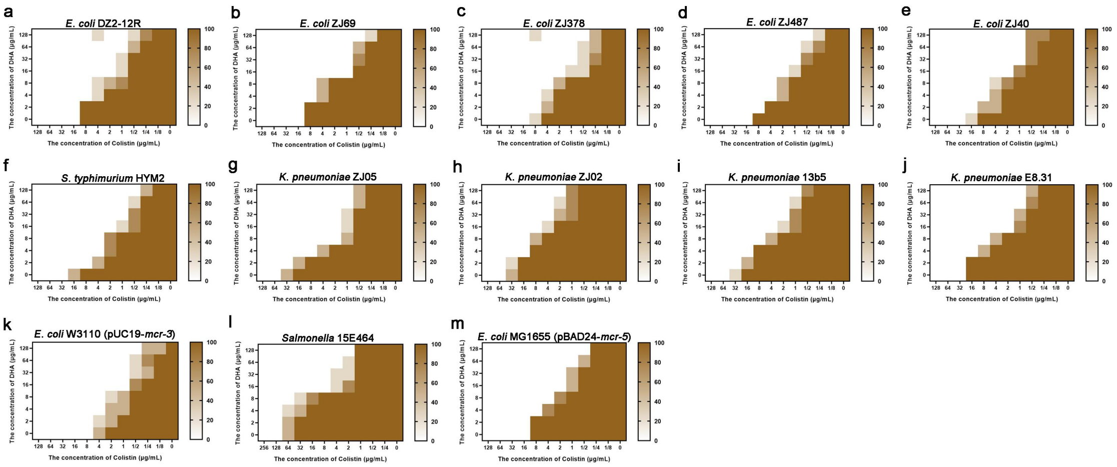

Supplementary Fig 3. The checkerboard MIC detection of DHA in combination with colistin against MCR-1/3/5-positive bacterial strains. a-m The checkerboard MICs were determined in quadruplicate, and used the color shade to indicate the probability of bacterial growth in the well. The more bacterial growth visible to the naked eye, the darker the color.

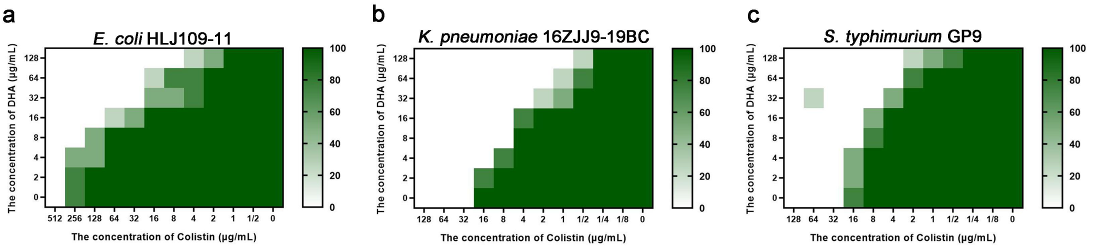

Supplementary Fig 4. The checkerboard MIC detection of DHA in combination with colistin against MCR-1-negative polymyxin-resistant bacterial strains. a-c The checkerboard MICs were determined in quadruplicate, and used the color shade to indicate the probability of bacterial growth in the well. The more bacterial growth visible to the naked eye, the darker the color.

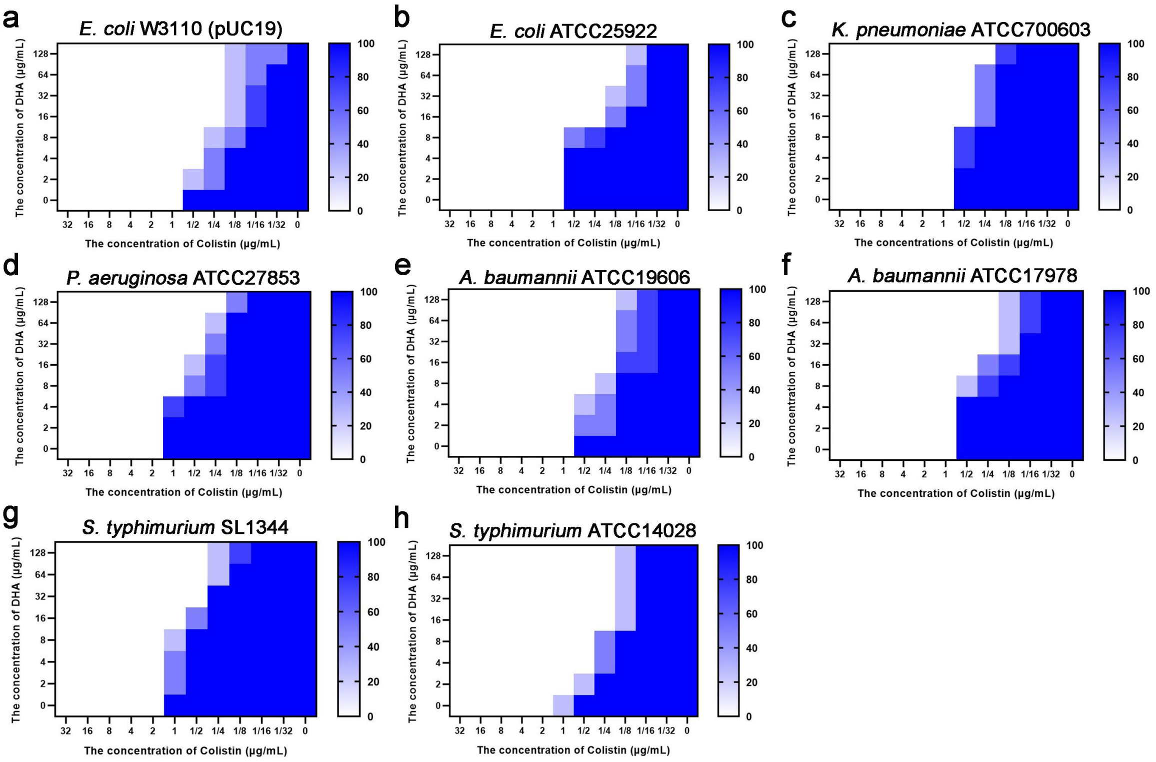

Supplementary Fig 5. The checkerboard MIC detection of DHA in combination with colistin against polymyxin-sensitive bacterial strains. a-h The checkerboard MICs were determined in quadruplicate, and used the color shade to indicate the probability of bacterial growth in the well. The more bacterial growth visible to the naked eye, the darker the color.

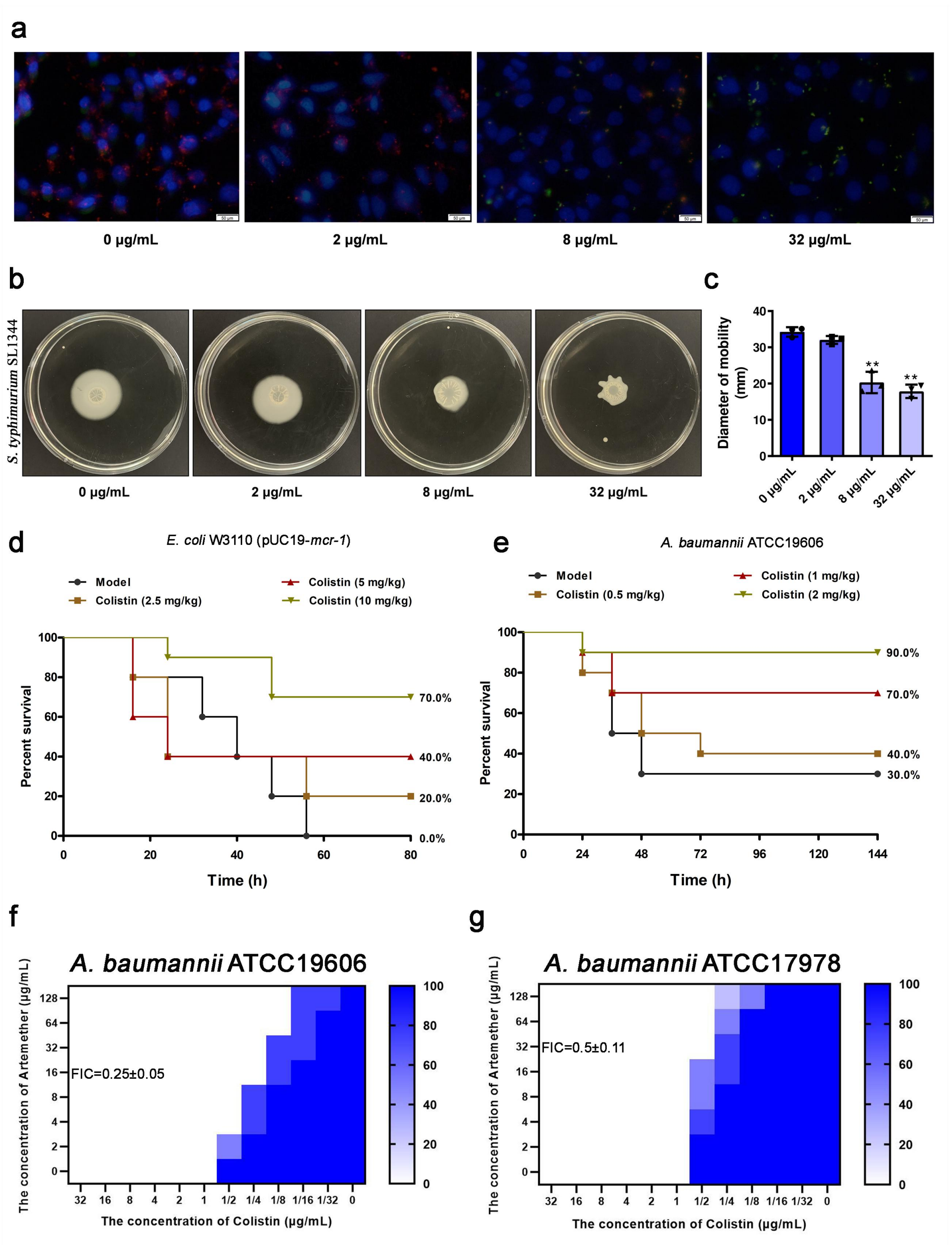

Supplementary Fig 6. a Immunofluorescence microscopy detection of DHA on *S.typhimurium* SL1344 (MCR-1-negative) invasion of HeLa cells (scale bar = 50 μm). **b, c** The motility of *S. typhimurium* SL1344 on agar plates was photographed, and the mobility diameter was measured. **d, e** Effective dose screening of colistin for the treatment of *E. coli* W3110 (pUC19-*mcr-1*) or *A. baumannii* ATCC19606

infection(n=10, per group). **f, g** The checkerboard MIC detection of artemether in combination with colistin against *A. baumannii* ATCC19606 and *A. baumannii* ATCC17978. The checkerboard MICs were determined in quadruplicate, and used the color shade to indicate the probability of bacterial growth in the well. The more bacterial growth visible to the naked eye, the darker the color.

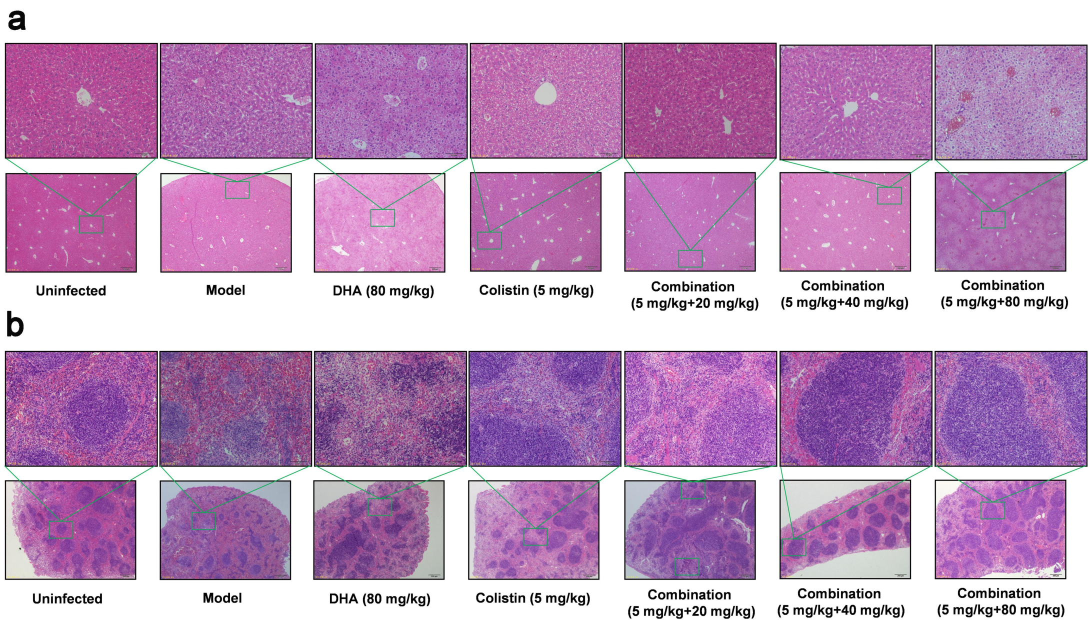

Supplementary Fig 7. Histopathology of the liver and spleen of *E. coli* W3110 (pUC19-*mcr-1*) infected mice after administration. **a, b** Histopathology of the liver (**a**) and spleen (**b**) of *E. coli* W3110 (pUC19-*mcr-1*) infected mice after administration (scale bar = 200 μm). Fewer pathological changes in the liver and spleen were noted after low-combined administration (5 mg/kg +20 mg/kg) and medium-combined administration (5 mg/kg +40 mg/kg) than after the administration of either agent

alone. However, high-dose combined administration (5 mg/kg +80 mg/kg) and DHA injection (80 mg/kg) alone appeared to have a significant lesion on the liver with hyperemia and cellular deformation.

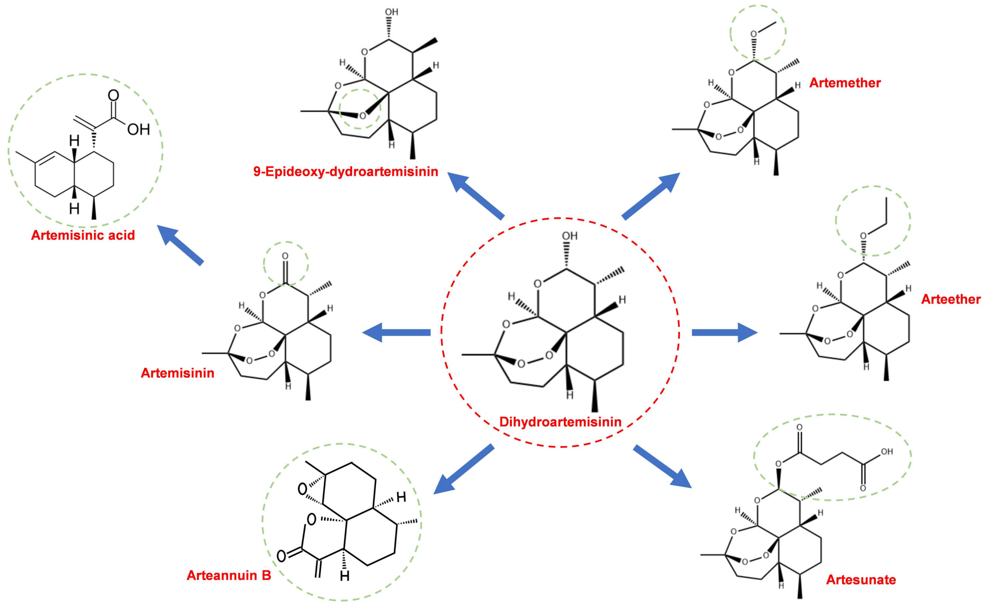

Supplementary Fig 8. The relationship between the molecular structures of artemisinin derivatives. Artesunate, artemether and arteether are extremely similar to DHA. 9-Epideoxy-dydroartemisinin and artemisinin have the second highest similarity with DHA.

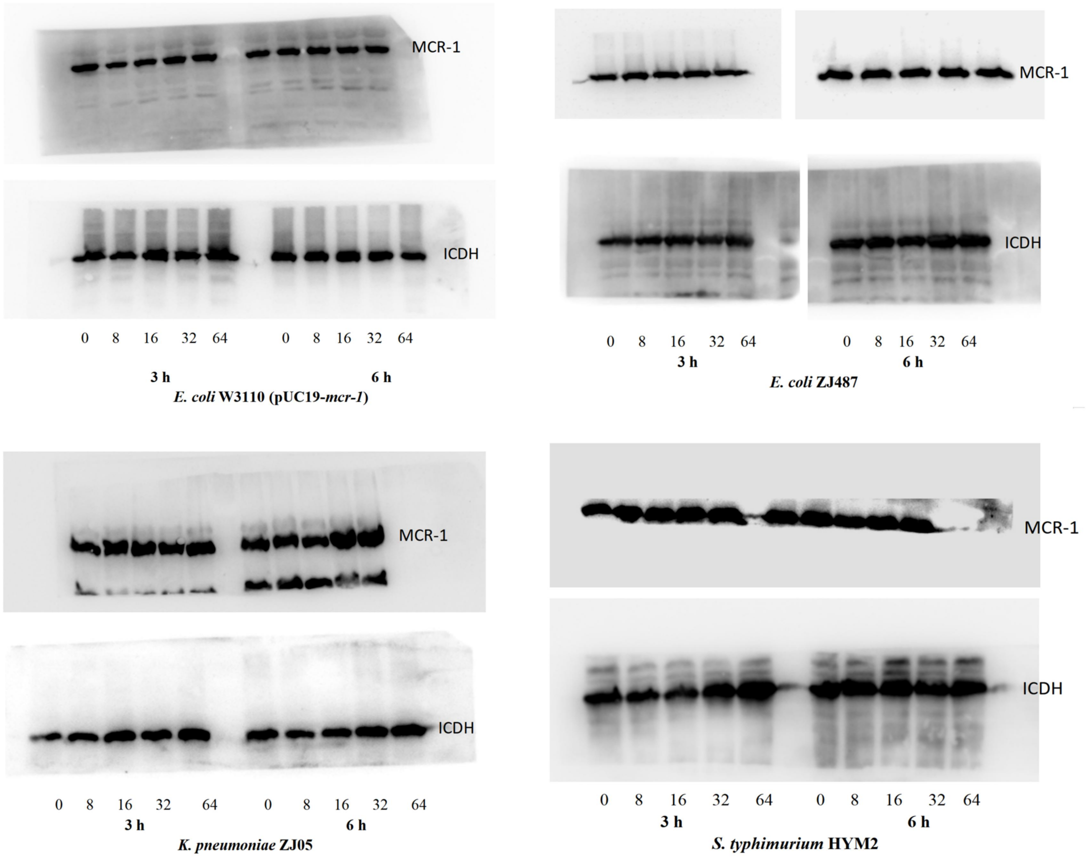

Supplementary Fig 9. Uncropped western blotting images. The effect of different concentrations of DHA (0, 8-64 μg/mL) treatment on the expression of MCR-1 in different strains of bacteria in 3h or 6 h.

Supplementary Table 1. MIC values of colistin and DHA combination therapy for each of the tested bacterial isolates.

Species	Source/Description	MIC (μg/mL)		*mcr-1/3/5* confirmation	FICs index
		Alone	Combination		
E. coli W3110 (pUC19-*mcr-1*)	Laboratory strain (carried a *mcr-1* gene that originated from *K. pneumoniae* ZJ05)	8.00±0.00	0.50±0.00	+	0.09±0.00
E. coli W3110 (pUC19)	Laboratory strain	1.00±0.00	0.14±0.07	-	0.17±0.07
E. coli DZ2-12R	Chicken cloacae	16.00±0.00	1.25±0.43	+	0.11±0.03
E. coli ZJ69	Human urine	16.00±0.00	1.00±0.00	+	0.09±0.00
E. coli ZJ378	Human feces	10.00±3.46	0.88±0.65	+	0.11±0.03
E. coli ZJ487	Human intra-abdominal fluid (*bla*$_{NDM-1}$-carrying）	16.00±0.00	1.25±0.43	+	0.11±0.03
E. coli ZJ40	Remote tertiary care hospital (*mcr-1* located in chromosome)	20.00±6.93	1.75±0.43	+	0.13±0.03
S. typhimurium HYM2	Animal farm	24.00±8.00	1.13±0.54	+	0.08±0.02
K. pneumoniae ZJ05	Remote tertiary care hospital	48.00±16.00	1.25±0.43	+	0.06±0.01
K. pneumoniae ZJ02	Remote tertiary care hospital	48.00±16.00	5.00±1.73	+	0.14±0.03
K. pneumoniae 13b5	Chicken cloacae	40.00±13.86	2.25±1.09	+	0.09±0.01
K. pneumoniae E8.31	Chicken cloacae	32.00±0.00	1.75±0.43	+	0.09±0.01
E. coli W3110 (pUC19-*mcr-3*)	Laboratory strain (carried a *mcr-3* gene)	6.00±2.00	0.88±0.22	+	0.19±0.05
E. coli MG1655 (pBAD24-*mcr-5*)	Laboratory strain (carried a *mcr-5* gene)	16.00±0.00	1.50±0.50	+	0.13±0.03
Salmonella 15E464	I4,5,12:i:- (carried a *mcr-3* gene)	96.00±32.00	4.50±2.18	+	0.08±±0.02

E. coli HLJ109-11	*pmr* B mutation (Polymyxin-resistant *mcr*-negative)	448.00±110.85	64.00±39.19	-	0.17±0.07
K. pneumoniae 16ZJJ9-19BC	Chicken cloacae (Polymyxin-resistant *mcr*-negative)	32.00±0.00	7.00±1.73	-	0.25±0.05
S. typhimurium GP9	Animal farm (Polymyxin-resistant *mcr*-negative)	24.00±8.00	12.00±4.00	-	0.53±0.00
E. coli ATCC25922	Laboratory strain	1.00±0.00	0.19±0.06	-	0.22±0.06
K. pneumoniae ATCC700603	Laboratory strain	1.00±0.00	0.38±0.13	-	0.41±0.13
P. aeruginosa ATCC27853	Laboratory strain	2.00±0.00	0.56±0.27	-	0.31±0.14
A. baumanii ATCC19606	Laboratory strain	1.00±0.00	0.11±0.03	-	0.14±0.03
A. baumanii ATCC17978	Laboratory strain	1.00±0.00	0.34±0.16	-	0.38±0.16
S. typhimurium ATCC14028	Laboratory strain	1.25±0.43	0.16±0.05	-	0.16±0.00
S. typhimurium SL1344	Derived from the virulent strain SL1344	2.00±0.00	0.75±0.25	-	0.41±0.13

All MICs were determined in quadruplicate. The concentration of DHA was 16 μg/mL in all bacteria. And the data were presented as the mean ± standard deviation. "+" represents *mcr-1*-positive species, "-" represents *mcr-1*-negative species.

Supplementary Table 2. The primers of all the point mutations for MCR-1

Primer names	Oligonucleotide primer sequence (5'–3')
MCR-1-WT	F: CGCGGATCCTGTATGGGATTGCGCAATGATT
	R: CCGCTCGAGTCAGCGGATGAATGCGGTGCGGTCTTT
MCR-1-MET-336-Ala	F: GGACTCAAAAGGCGTGGCGGATAAGCTGCCAAAAG
	R: CTTTTGGCAGCTTATCCGCCACGCCTTTTGAGTCC
MCR-1-ASP-337-Ala	F: CAAAAGGCGTGATGGCGAAGCTGCCAAAAGC
	R: GCTTTTGGCAGCTTCGCCATCACGCCTTTTG
MCR-1-PRO-340-Ala	F: CGTGATGGATAAGCTGGCGAAAGCGCAATTTGCCG
	R: CGGCAAATTGCGCTTTCGCCAGCTTATCCATCACG
MCR-1-GLN-343-Ala	F: GATAAGCTGCCAAAAGCGGCGTTTGCCGATTATAAATCC
	R: GGATTTATAATCGGCAAACGCCGCTTTTGGCAGCTTATC
MCR-1-LYS-348-Ala	F: GCAATTTGCCGATTATGCGTCCGCGACCAACAACG
	R: CGTTGTTGGTCGCGGACGCATAATCGGCAAATTGC
pUC19 vector	F: GGAGGATCCGGATCCATCGTTCAAAC
	R: ATCGAATTCATCGATTACTAGTAACATAG

[a] Mutated codons are underlined.

Supplementary Table 3. Composition of dihydroartemisinin injection

Composition	Content	Manufacturer	Molecular weight
N-N-Dimethylacetamide	30 mL	Finar Ltd., Gujarat, India.	87.12
Castor oil	69 mL	Hunan Er-Kang Pharmaceutical Co., Ltd.,Hunan, China	/
Benzyl alcohol	1 mL	Tianjin Huadong Reagent Factory, Tianjin, China	108.14
Dihydroartemisinin	1 g	Chengdu Deruike Biotechnology Co., Ltd. Chengdu, China	284.35
