# Supplementary material for: Commercialized artemisinin derivatives combined with colistin protect against critical Gram-negative bacterial infection
Source: Commun Biol. 2022 Sep 8;5:931. doi: 10.1038/s42003-022-03898-5 (PMC9458715; doi:10.1038/s42003-022-03898-5)
Supplement: Supplementary file 4 — Reporting summary [file 42003_2022_3898_MOESM4_ESM.pdf]

nature portfolio

Corresponding author(s): COMMSBIO-22-1183A

Last updated by author(s): Jul 10, 2022

Reporting Summary

Nature Portfolio wishes to improve the reproducibility of the work that we publish. This form provides structure for consistency and transparency in reporting. For further information on Nature Portfolio policies, see our Editorial Policies and the Editorial Policy Checklist.

Statistics

For all statistical analyses, confirm that the following items are present in the figure legend, table legend, main text, or Methods section.

n/a	Confirmed	
☐	☒	The exact sample size (*n*) for each experimental group/condition, given as a discrete number and unit of measurement
☐	☒	A statement on whether measurements were taken from distinct samples or whether the same sample was measured repeatedly
☐	☒	The statistical test(s) used AND whether they are one- or two-sided<br>*Only common tests should be described solely by name; describe more complex techniques in the Methods section.*
☒	☐	A description of all covariates tested
☒	☐	A description of any assumptions or corrections, such as tests of normality and adjustment for multiple comparisons
☐	☒	A full description of the statistical parameters including central tendency (e.g. means) or other basic estimates (e.g. regression coefficient) AND variation (e.g. standard deviation) or associated estimates of uncertainty (e.g. confidence intervals)
☒	☐	For null hypothesis testing, the test statistic (e.g. *F*, *t*, *r*) with confidence intervals, effect sizes, degrees of freedom and *P* value noted<br>*Give P values as exact values whenever suitable.*
☒	☐	For Bayesian analysis, information on the choice of priors and Markov chain Monte Carlo settings
☐	☒	For hierarchical and complex designs, identification of the appropriate level for tests and full reporting of outcomes
☒	☐	Estimates of effect sizes (e.g. Cohen's *d*, Pearson's *r*), indicating how they were calculated

Our web collection on statistics for biologists contains articles on many of the points above.

Software and code

Policy information about availability of computer code

Data collection	We use Microsoft Excel to collect the data in this study.
Data analysis	Statistical analysis was performed using SPSS version 19.0 and GraphPad Prism 7.0.

For manuscripts utilizing custom algorithms or software that are central to the research but not yet described in published literature, software must be made available to editors and reviewers. We strongly encourage code deposition in a community repository (e.g. GitHub). See the Nature Portfolio guidelines for submitting code & software for further information.

Data

Policy information about availability of data

All manuscripts must include a data availability statement. This statement should provide the following information, where applicable:

- Accession codes, unique identifiers, or web links for publicly available datasets
- A description of any restrictions on data availability
- For clinical datasets or third party data, please ensure that the statement adheres to our policy

All data included in this study are available upon request by contact with the corresponding author.

Field-specific reporting

Please select the one below that is the best fit for your research. If you are not sure, read the appropriate sections before making your selection.

☒ Life sciences ☐ Behavioural & social sciences ☐ Ecological, evolutionary & environmental sciences

For a reference copy of the document with all sections, see nature.com/documents/nr-reporting-summary-flat.pdf

Life sciences study design

All studies must disclose on these points even when the disclosure is negative.

Sample size	For mouse infection model assays, the mice (n=17, per group) were intraperitoneally infected with E. coli W3110 (pUC19-mcr-1) to cause a systemic infection.The mouse (n=16, per group) intraperitoneal infection model A. baumannii ATCC19606 was also used to confirm the synergistic effect of colistin and artemether injection. For S. typhimurium infection analysis, the mice (n=15, per group) were housed for 5 days with water containing streptomycin (5 mg/mL) before infection and were orally gavaged with S. typhimurium HYM2.For bacterial loading analysis, weight analysis and morbidity assays, the mice (n=10, per group) were intraperitoneally infected with E. coli W3110 (pUC19-mcr-1).
Data exclusions	No data were exccluded from the analyses.
Replication	The experiments were repeated at least three times independently, and representative data are displayed.
Randomization	All samples were randomly assigned to different groups. Such as the experimental mice were first mixed together then randomly grabbed and divided into different groups before treatment.
Blinding	The investigators were blinded to group allocation during data collection and analysis.

Reporting for specific materials, systems and methods

We require information from authors about some types of materials, experimental systems and methods used in many studies. Here, indicate whether each material, system or method listed is relevant to your study. If you are not sure if a list item applies to your research, read the appropriate section before selecting a response.

Materials & experimental systems

n/a	Involved in the study	
☒	☐	Antibodies
☐	☒	Eukaryotic cell lines
☒	☐	Palaeontology and archaeology
☐	☒	Animals and other organisms
☒	☐	Human research participants
☒	☐	Clinical data
☒	☐	Dual use research of concern

Methods

n/a	Involved in the study	
☒	☐	ChIP-seq
☒	☐	Flow cytometry
☒	☐	MRI-based neuroimaging

Eukaryotic cell lines

Policy information about cell lines

Cell line source(s)	J774 cells, Vero cells and HeLa cells in current study were obtained from the American Type Culture Collection (ATCC).
Authentication	Our laboratory uses the gold standard STR method for cell identification.
Mycoplasma contamination	Confirmed free from mycoplasma.
Commonly misidentified lines (See ICLAC register)	HeLa cells, for the invasion ability of S. typhimurium.

Animals and other organisms

Policy information about studies involving animals; ARRIVE guidelines recommended for reporting animal research

Laboratory animals	6-8-week old female BALB/c mice (20 ± 2 g) were obtained from the Liaoning Changsheng Biotechnology Co., Ltd.
Wild animals	Our study did not involve wild animals.

Field-collected samples	Our study did not involve samples collected form Field.
Ethics oversight	Animal experiments were approved by the Animal Care and Use Committee of Jilin University (No. ALKT202102003) and were operated following the guidelines of this committee.

Note that full information on the approval of the study protocol must also be provided in the manuscript.
